# Down-Regulation of eIF4GII by miR-520c-3p Represses Diffuse Large B Cell Lymphoma Development

**DOI:** 10.1371/journal.pgen.1004105

**Published:** 2014-01-30

**Authors:** Krystyna Mazan-Mamczarz, X. Frank Zhao, Bojie Dai, James J. Steinhardt, Raymond J. Peroutka, Kimberly L. Berk, Ari L. Landon, Mariola Sadowska, Yongqing Zhang, Elin Lehrmann, Kevin G. Becker, Rita Shaknovich, Zhenqiu Liu, Ronald B. Gartenhaus

**Affiliations:** 1 Marlene & Stewart Greenebaum Cancer Center, Department of Medicine, University of Maryland, Baltimore, Maryland, United States of America; 2 Department of Pathology, University of Maryland, Baltimore, Maryland, United States of America; 3 Gene Expression and Genomics Unit, National Institute of Aging, National Institutes of Health, Baltimore, Maryland, United States of America; 4 Veterans Administration Medical Center, Baltimore, Maryland, United States of America; 5 Pathology and Laboratory Medicine, Weill Cornell Medical College, New York, New York, United States of America; University of Washington, United States of America

## Abstract

Deregulation of the translational machinery is emerging as a critical contributor to cancer development. The contribution of microRNAs in translational gene control has been established however; the role of microRNAs in disrupting the cap-dependent translation regulation complex has not been previously described. Here, we established that elevated miR-520c-3p represses global translation, cell proliferation and initiates premature senescence in HeLa and DLBCL cells. Moreover, we demonstrate that miR-520c-3p directly targets translation initiation factor, eIF4GII mRNA and negatively regulates eIF4GII protein synthesis. miR-520c-3p overexpression diminishes cells colony formation and reduces tumor growth in a human xenograft mouse model. Consequently, downregulation of eIF4GII by siRNA decreases translation, cell proliferation and ability to form colonies, as well as induces cellular senescence. *In vitro* and *in vivo* findings were further validated in patient samples; DLBCL primary cells demonstrated low miR-520c-3p levels with reciprocally up-regulated eIF4GII protein expression. Our results provide evidence that the tumor suppressor effect of miR-520c-3p is mediated through repression of translation while inducing senescence and that eIF4GII is a key effector of this anti-tumor activity.

## Author Summary

Control of gene expression on the translational level is critical for proper function of major cellular processes and deregulation of translation can promote cellular transformation. Emerging actors in this post-transcriptional gene regulation are small non-coding RNAs referred to as microRNAs (miRNAs). We established that miR-520c-3p represses tumor growth through the repression of eIF4GII, a major structural component of the translation initiation complex. Since translation of most cellular mRNAs is primarily regulated at the level of initiation, this node is becoming a potential target for therapeutic intervention. Identified in this study, tumor suppressor function of miR-520c-3p is mediated through the inhibition of translational factor eIF4GII, resulting in the repression of global translational machinery and induction of senescence in tumor cells. While aging and senescence has been shown to be associated with reduced translation the linkage between translational deregulation and senescence in malignant cells has not been previously described. Lending further clinical significance to our findings, we were able to demonstrate that primary DLBCL samples had elevated levels of eIF4GII while having reciprocally low miR-520c-3p expression.

## Introduction

Control of gene expression at the level of mRNA translation is a crucial step that regulates proper function of major cellular processes such as cell proliferation, growth, differentiation, apoptosis, stress response, and tumorigenesis. A large body of recent research indicates that the deregulation of mRNA translation can promote cellular transformation and a malignant phenotype [1]–[4]. Cellular senescence, resulting in permanent arrest of cell growth is emerging as an intrinsic tumor suppressive mechanism [5]. While it has been established that aging and senescence is associated with lower rates of mRNA translation the linkage between translation deregulation and senescence in malignant cells is poorly described. Therefore, understanding the translational regulation in the framework of senescence program in tumor cells may provide an important approach in cancer therapy.

Translation of most mRNAs is primarily regulated at the level of initiation, a process that requires the protein complex known as eukaryotic initiation factor 4F (eIF4F), consisting of three proteins: cap-binding protein eIF4E, scaffolding protein eIF4G, and ATP-dependent RNA helicase eIF4A [6], [7]. The aberrant expression of eIF4F components has been shown to be involved in many cancers, particularly in B-cell lymphoma [8]–[10]. Thus, targeting of the translation initiation complex is emerging as a potential cancer therapy [11]–[13]. The eukaryotic translation initiation factor 4, gamma (eIF4G) is expressed in mammalian cells in the form of the two homologs, eIF4GI and eIF4GII [14], [15]. Recent studies have found that eIF4GI and eIF4GII despite the biochemical and functional similarities can fulfill different roles in mammalian cells [7]. Elevated eIF4GI levels have been shown to correlate with a malignant cell transformation [16]–[19]. However, little is currently known about eIF4GII expression and its function in translation initiation.

MicroRNAs (miRNAs) are endogenous regulatory RNA molecules that modulate protein expression based on sequence complementation with their target messenger RNAs (mRNAs) [20]–[24]. Given that these small regulatory molecules are frequently deregulated in various human cancers, they have become potential candidates as biomarkers and therapeutic intervention. Gene expression profiling studies and bioinformatics analysis have discovered numerous miRNAs differentially expressed in several types of human cancers, including B-cell malignancies [25], [26]. Among the miRNAs expressed by hematopoietic cells, several were implicated in different types of human B-cell lymphomas [27], [28] and suggest a promising option for lymphoma/leukemia therapy [29]–[31]. However, the mechanisms by which these small regulatory RNAs deregulation contribute to lymphomagenesis have not been well established. Therefore, the identification of the genes and pathways directly targeted by microRNAs remains to be fully elucidated.

Herein, we discovered that elevated expression of miR-520c-3p results in decreased global translation and cell proliferation, and induced premature senescence in HeLa and diffuse large B-cell lymphoma (DLBCL). Furthermore, miR-520c-3p negatively regulates the protein synthesis of eIF4G3/eIF4GII (known from here out as eIF4GII) through the direct targeting of its mRNA. Overexpression of miR-520c-3p resulted in diminished clonogenic capacity of DLBCL cells and reduced tumor growth in a human lymphoma xenograft mouse model. Importantly, we found that DLBCL cell lines and patient samples expressed reduced levels of miR-520c-3p and elevated levels of eIF4GII as compared to normal donor B-cell lymphocytes. Our findings presented here provide a previously unknown tumor suppressor function for miR-520c-3p elicited by decreasing eIF4GII expression, resulting in repressed global protein synthesis and induction of premature cellular senescence.

## Results

### Overexpression of miR-520c-3p Decreased Global Translation and Proliferation and Induced Senescence in HeLa and DLBCL Cells

The contribution of microRNAs in control of post-transcriptional gene regulatory events in human cancers is becoming increasingly evident. In a recent survey identifying the microRNAs associated with elevated levels of the oncoprotein MCT-1 (data not shown), we found that overexpression of miR-520c-3p greatly repressed global protein synthesis, suggesting that miR-520c-3p could contribute broadly to regulating protein abundance. Given that miR-520c was found to have decreased expression in the series of DLBCL cases used for miRNA profiling [32] and deregulation of protein synthesis has been recognized as a critical part of cancer development [33]–[35] we focused on miR-520c-3p function in human cancers with a particular interest in DLBCL. The human cervical carcinoma HeLa cell line was used for the mechanistic studies because these cells can be easily transfected at a very high efficiency, an important requirement for such experiments.

**Figure 1:**
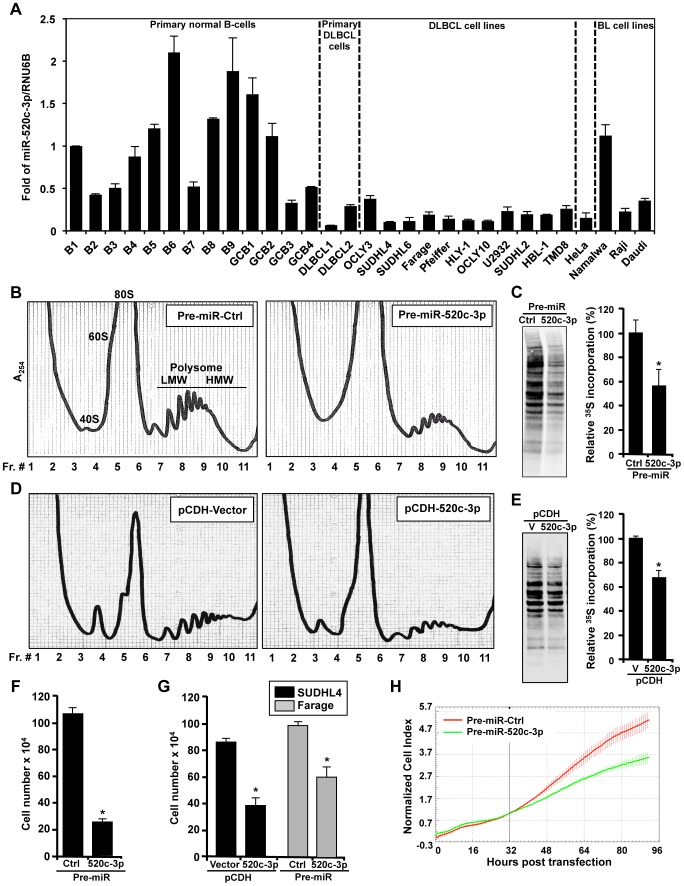
miR-520c-3p is downregulated in DLBCL cells and affects global gene translation and cell proliferation. (A) Total RNA from normal B-cells, DLBCL, BL, and HeLa cell lines were used to measure miR-520c-3p abundance by RT-qPCR. Graphs represent the means and SEM from repeats of three independent assays. (B) 48 h after transfection with Pre-miR-Ctrl or Pre-miR-520c-3p HeLa cells were fractionated through sucrose gradients. Absorbance at 254 nm was used to identify the fractions containing ribosomal subunits 40S and 60S, monosomes 80S, and polysomes LMW and HMW (low- and high-molecular weight). (C) 48 h after transfection as described in (B) HeLa cells incubated for 20 min with ^35^S-labeled amino acids. Lysate aliquots corresponding to the same cell numbers were size fractionated by SDS-PAGE, transferred onto PVDF membranes, and visualized using a PhosphorImager. The ^35^S-amino acid incorporation was quantified (graph) and is presented as percent signal intensity relative to control transfection group. (D) Polysome profiles obtained 48 h after transduction of SUDHL4 cells with either empty vector (pCDH-Vector; V) or vector overexpressing miR-520c-3p (pCDH-520c-3p). (E) SUDHL4 cells were transduced as described in (D) and radiolabeled 48 h later as described in (C). (F) 72 h after transfection of HeLa cells as described in (B) cell numbers were counted using a hemocytometer. (G) 72 h after transfection cells as described in Materials and Methods cell numbers were counted using a hemocytometer. (H) After transfection as in (B), HeLa cell proliferation in real-time was monitored by the xCELLigence System. The data are representative of at least three independent experiments. In the graphs (A), (C), (E), (F) and (G) the means and SEM are shown. * indicates p<0.05.

**Figure 2:**
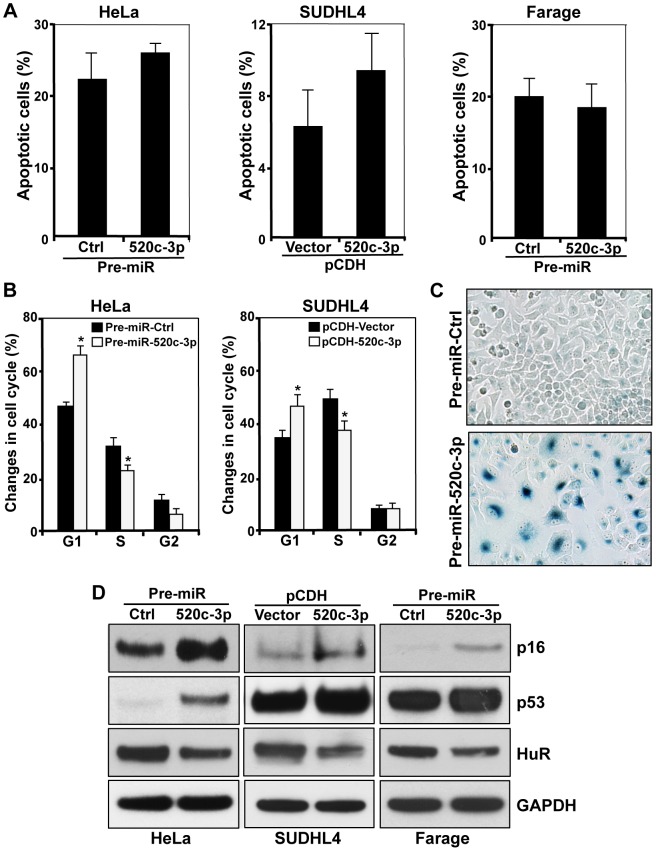
Influence of miR-520c-3p on the senescent phenotype. Cells were transfected/transduced as described in Materials and Methods. (A) Six days after transfection the percentage of apoptotic cells was determined by flow cytometry. (B) 72 h after transfection cells were stained with PI and subjected to cell cycle analysis. (C) HeLa cells were tested for β-galactosidase activity six days after transfection. Representative pictures are shown. (D) Six days after transfection the levels of p16, p53 and HuR were assessed by Western blot analysis. GAPDH served as a loading control. The data are representative of at least three independent experiments. In the graphs (A) and (B) the means and SEM are shown. * p<0.05.

First, we explored the abundance of miR-520c-3p in several primary normal B-cells, primary DLBCL cells, cultured DLBCL and Burkitt Lymphoma (BL) cell lines by RT-qPCR analysis. As shown in Figure 1A, miR-520c-3p levels were consistently lower in both primary DLBCL samples and DLBCL cell lines as compared to normal B-cells. Forty-eight and seventy-two hours after transfection to elevate the levels of miR-520c-3p, significant reduction in the global protein synthesis in miR-520c-3p overexpressing cells relative to the control cultures was demonstrated by polysome distribution profiles and incorporation of ^35^S-labeled amino acids into nascent protein in HeLa (Figure 1B and C, Figure S1A–C) and DLBCL (Figure 1D and E, Figure S1D–F) cell populations. Inversely, reduction of miR-520c-3p expression by miRZip-520c-3p transduction increased global protein synthesis levels. However, these observed differences were not as dramatic as the reduction after overexpression of mir-520c-3p (Figure S2A–D). This attenuated impact was likely due to the already low levels of miR-520c-3p present in the cells. Consistent with the repressed translation, the cell proliferation was reduced in all studied cell lines as fewer cells were observed in the miR-520c-3p upregulated populations compared to the control miRNA groups when examined by cell counting (Figure 1F and G) and measured in real-time by the xCELLigence System in HeLa cells (Figure 1H). Upon miR-520c-3p overexpression, all studied cell lines showed only slight or no increase in cell apoptosis, as measured by Annexin V staining (Figure 2A). Furthermore, flow cytometry analysis showed that seventy-two hours after transfection cells with increased miR-520c-3p expression delayed passage through G_1_ phase as compared to control-transfected populations (Figure 2B, Figure S3A and B). Interestingly, we discovered that HeLa cells overexpressing miR-520c-3p were enlarged, displayed a distinctive flat morphology, and had numerous vacuoles, which is characteristic of senescent cells. The induction of a senescent phenotype triggered by increased levels of miR-520c-3p was further supported by the enhanced activity of the senescence marker ß-galactosidase (Figure 2C) and was confirmed by other hallmarks of senescence such as elevated p16 and p53 protein levels and decreased HuR expression [36] in HeLa and DLBCL cells (Figure 2D). Altogether, these data indicate that overexpression of miR-520c-3p inhibited global translation and cell proliferation, and induced cellular senescence in cancer cells.

### Elevated Levels of miR-520c-3p Decreased Translation of Its Target Genes

**Figure 3:**
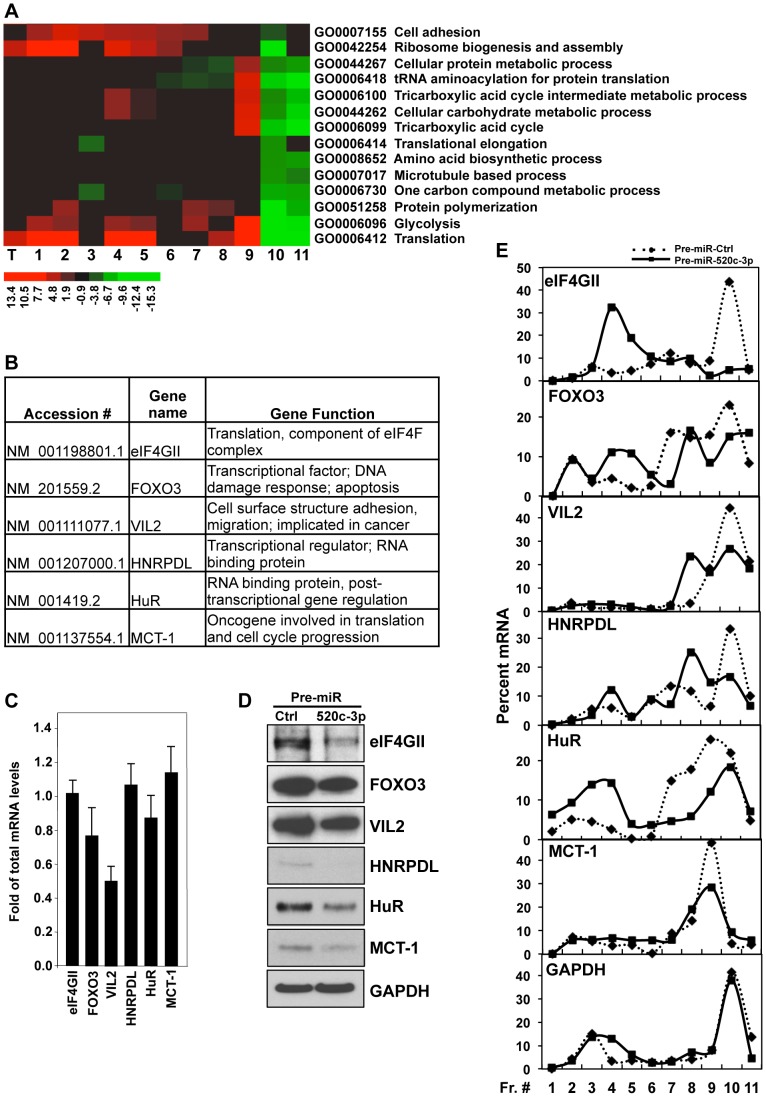
Analysis of microarray data in HeLa cells. (A) Functional categories of total and polysome associated mRNAs in Pre-miR-520c-3p compared to Pre-miR-Ctrl transfected cells. Heat map represents GO annotations with the most altered values in sucrose gradient fractions. Top 100 categories are illustrated in Figure S4. T indicates total RNA; lanes 1 through 11 represent RNA from sucrose fractions of increasing molecular weight. (B and C) The levels of total mRNAs of validated genes were measured by RT-qPCR in cells transfected with Pre-miR-520c-3p compared to Pre-miR-Ctrl. Graphs represent the means and SEM from three repeats of three independent assays. (D) Protein levels of miR-520c-3p target genes were measured by Western blotting. GAPDH was used as a loading control. (E) Cells were fractionated through sucrose gradients and the relative distribution of selected miR-520c-3p target mRNA (and housekeeping GAPDH mRNA) was studied by RT-qPCR analysis of RNA in each of 11 gradient fractions. Data are representative of three independent experiments.

To better understand the molecular mechanisms whereby miR-520c-3p elicits global translational repression and to identify miR-520c-3p targets and pathways involved in this regulation, we performed microarray analysis of total mRNA and mRNA isolated from polysomal fractions (translational profiling). Significantly altered genes were further analyzed for representation of Gene Ontology (GO) terms to identify key biologically functional categories that were significantly changed in miR-520c-3p overexpressed compared to control transfected cells. Noticeably, GO analysis revealed mainly translation and translation related categories with greatest representations of genes which had decreased protein synthesis (lowest values in the most actively translating fractions [fractions 10–11], and highest values in the non-translating and low-translating fractions of the gradient [fractions 1–7]) in miR-520c-3p overexpressed versus control cells (Figure 3A, Figure S4). Additionally, genes affected by miR-520c-3p overexpression in the most translationally active polysomal fraction 10 were put in the context of the known molecular interactions by using Ingenuity Pathways Analysis (IPA). IPA revealed that the top five networks, with the highest number of involved genes, were molecular functions linked to RNA post-transcriptional modification, cellular development, cellular growth and proliferation, cell cycle, and cancer (Table S1). The effect of miR-520c-3p overexpression was furthermore verified for several mRNAs (Figure 3B) chosen from the most significantly altered in microarray data genes (Table S2), which were computationally predicted as direct targets of miR-520c-3p (by miRanda and Microcosm database) and, functionally represented above ranked pathways found by GO and IPA analysis. By comparing miR-520c-3p upregulated to control transfected cells by RT-qPCR analysis, we observed no influence of miR-520c-3p on total mRNA levels of most of the validated miR-520c-3p target transcripts (Figure 3C). Instead, we found that increased miR-520c-3p levels lowered protein expression of studied target genes (Figure 3D).

Given that microRNAs have been shown to influence translation as well as message stability [37], we investigated both potential post-transcriptional mechanisms (Figure 3E, Figure S5). We found only one message (Vil2 mRNA) to be affected on both the stability and translational level by miR-520c-3p. As shown in Figure 3E, corresponding with protein levels, we found decreased levels of all the validated transcripts in the actively translating fractions of the gradient (fractions 8–11), and elevated levels in the non-translating and low-translating fractions of the gradient (fractions 1–7) in groups transfected with miR-520c-3p. These differences were not observed when testing the distribution of the housekeeping GAPDH mRNA. The above data confirm that miR-520c-3p targets and decreases expression of many important genes involved in translational control, cell proliferation and cancer development.

### eIF4GII Is a Specific Target of miR-520c-3p

**Figure 4:**
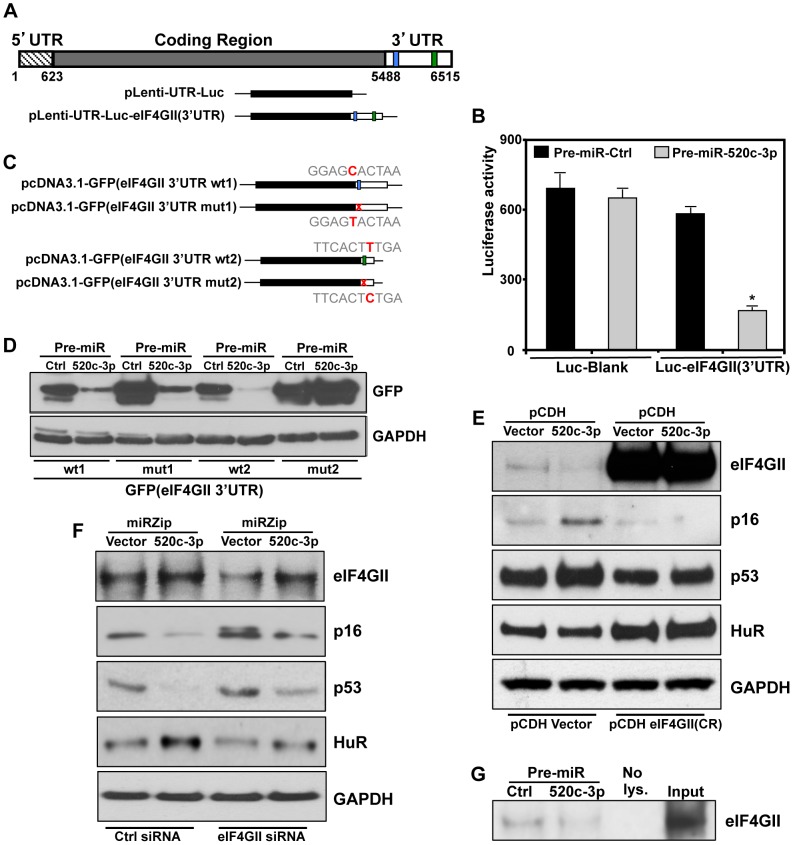
eIF4GII is a specific target of miR-520c-3p. (A) Schematics of eIF4GII mRNA depicting the miR-520c-3p predicted target sites in 3′-untranslated region (3′UTR) and luciferase reporter construct bearing either no eIF4GII mRNA sequences or eIF4GII 3′UTR. (B) Plasmids pLenti-UTR-Luc-eIF4GII(3′UTR) or pLenti-UTR-Luc-Blank were cotransfected with Pre-miR-Ctrl or Pre-miR-520c-3p; the levels of luciferase activity were measured 48 h after cotransfection. Average and SD of three independent experiments are shown. * p<0.05. (C) Schematic of GFP reporter constructs bearing segments with predicted miR-520c-3p sites either wild type (wt) or with mutated (mut) seed sequences on eIF4GII 3′UTR. (D) 48 h after cotransfection of the plasmids from (C) with Pre-miR-Ctrl or Pre-miR-520c-3p in HeLa cells, the expression of GFP in each transfection group were analyzed by Western blot. (E) Six days after cotransfection of SUDHL4 cells with pCDH Vector or pCDH-eIF4GII(CR) along with either pCDH-Vector or pCDH-520c-5p, Western blot was performed. (F) Western blot analysis of eIF4GII, p16, p53, and HuR expression six days after co-transfection HeLa cells with Ctrl siRNA or eIF4GII siRNA along with either miRZip-Vector or miRZip-520c-3p. GAPDH was used as a loading control. (G) At 48 h after transfection HeLa cells with Pre-miR-Ctrl or Pre-miR-520c-3p, pull-down reactions with 5′ 7 mG cap analog were performed. eIF4GII abundance was analyzed by Western blot. Input and 20 µg of whole cell lysates were included as a blotting control. All data are representative of three independent experiments.

Importantly, among the validated genes in which translation and protein levels were strongly decreased by miR-520c-3p was eukaryotic translation initiation factor 4 gamma, 3 (eIF4GII), one of the crucial components of the cap-dependent translation regulation complex eIF4F (Figure 3D and E). Therefore, we hypothesized that the influence of miR-520c-3p overexpression on global protein synthesis and cell phenotype might be mediated by eIF4GII. eIF4GII was predicted to have two miR-520c-3p binding sites in its 3′ untranslated region (3′UTR) (Figure 4A). The specific influence of miR-520c-3p on eIF4GII was initially confirmed by heterologous reporter constructs, which revealed diminished luciferase activity in the construct bearing the 3′ untranslated region (3′UTR) of eIF4GII mRNA suggesting that miR-520c-3p indeed repressed eIF4GII translation through its 3′UTR sequence (Figure 4B). Further, to corroborate that miR-520c-3p repressed eIF4GII translation specifically through the seed sites on its 3′UTR, heterologous GFP reporters were transfected in the conditions of either elevated miR-520c-3p or miR-Ctrl. These reporters were bearing segments with the predicted miR-520c-3p sites that were either wild type (wt) or with mutated (mut) seed region on eIF4GII 3′UTR (Figure 4C). As demonstrated in Figure 4D, increased miR-520c-3p levels efficiently reduced GFP wild type expression in both eIF4GII fragments, confirming miR-520c-3p's repressive ability on both studied sites. Mutation of the seed region 1 (mut1) only slightly increased GFP expression in miR-520c-3p overexpressing cells compared to the wild type. However, mutating the seed region 2 (mut2) in the eIF4GII 3′UTR strongly increased GFP levels in miR-520c-3p overexpressing populations. Significantly, GFP mRNA levels remained unchanged between transfection groups (Figure S6A). These data suggest that miR-520c-3p inhibits eIF4GII expression through two sites in eIF4GII 3′UTR but one site, seed region 1, may not be the true binding site for miR520c-3p. Instead seed region 2 is indeed functional; therefore mutation in site 2 prevents the downregulation of GFP protein.

The repression through the eIF4GII 3′UTR miR-520c-3p sites was additionally evaluated by rescue experiments. Transfection with miR-520c-3p efficiently repressed eIF4GII levels in vector transfected populations but did not affected the levels of overexpressed eIF4GII with deleted 3′UTR sequence, pCDH-eIF4GII(CR) (Figure 4E). Strikingly, overexpression of pCDH-eIF4GII(CR) also changed the levels of senescence markers and abolished miR-520c-3p triggered differences in their expression. On the contrary, knocking down miR-520c-3p with miRZip-520c-3p increased the expression of eIF4GII, was able to restore eIF4GII levels silenced by siRNA and modulated levels of studied senescence phenotype proteins (Figure 4F). Finally, we tested if miR-520c-3p overexpression affects the availability of eIF4GII for translation initiation and observed miR-520c-3p mediated reduction in the abundance of eIF4GII associated with the 5′ 7-methyl-GTP cap structure analog (Figure 4G). All together, these results strongly support the contention that the miR-520c-3p-elicited changes in protein translation and cell phenotype are indeed a consequence of modulated eIF4GII levels, via miR-520c-3p interaction with binding sites in the eIF4GII 3′UTR.

### Contribution of eIF4GII in the Phenotype Triggered by miR-520c-3p

**Figure 5:**
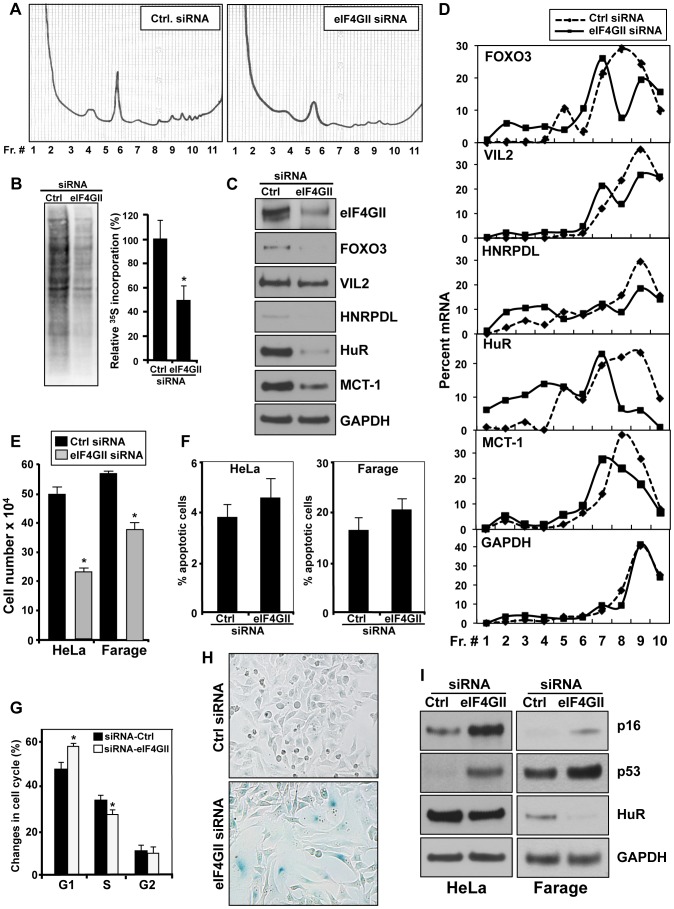
Influence of eIF4GII on gene translation, cell proliferation and the senescent phenotype. (A) 72 h after transfecting HeLa cells with either siRNA Ctrl or siRNA targeting eIF4GII, cell lysates were fractionated through 10–50% linear sucrose gradients, and the polysome distribution profiles were analyzed. (B) HeLa cells were transfected as described in (A) and nascent protein synthesis was assessed by incorporation of ^35^S-labeled amino acids. The ^35^S-amino acid incorporation was quantified and presented as percentage of signal intensity relative to control transfection (graph.). (C) Protein levels of miR-520c-3p target genes were measured by Western blotting 72 h after transfection of HeLa cells with either control siRNA or eIF4GII siRNA. GAPDH was used as a loading control. (D) Cells were transfected as described in (A) and the mRNA levels of selected miR-520c-3p target mRNAs and housekeeping GAPDH mRNA in each gradient fraction were measured by RT-qPCR and plotted as a percentage of the total mRNA levels in each sample. Data represent the average of three independent experiments showing similar results. (E) 72 h after transfection HeLa and Farage cells with either siRNA Ctrl or targeting eIF4GII siRNA, cell numbers were counted with a hemocytometer. (F) Six days after transfection as described in (E) cells were tested for apoptosis. (G) 72 h after transfection as described in (E) HeLa cells were stained with PI and subjected to cell cycle analysis. (H) β-galactosidase activity was measured in HeLa cells six days after transfection as described in (F) (I) Six days after transfections as described in Materials and Methods, the levels of p16, p53, and HuR in HeLa and Farage cells were assessed by Western blot analysis. GAPDH served as a loading control. Data are representative of at least three independent experiments. In the graphs (B), (E), (F) and (G) the means and SEM are shown. * p<0.05.

Given that mRNA translation is primarily regulated at the level of initiation and that eIF4G is a key scaffolding protein that forms a critical link between the mRNA cap structure and poly(A) tail during this process, we further postulated that the repression of the eIF4GII by miR-520c-3p might elicit a global mRNA translation inhibition upon miR-520c-3p overexpression. To test this possibility we silenced the eIF4GII protein levels by using eIF4GII-targeting siRNA. Indeed, knockdown of eIF4GII expression consistently repressed global translation as monitored by the global polysome distribution profiles (Figure 5A) and nascent protein synthesis assessed by incorporation of ^35^S-labeled amino acids (Figure 5B).

We also tested whether silencing of eIF4GII could affect the translation of specific mRNAs validated in Figure 3 as miR-520c-3p targets by monitoring the fraction of those mRNAs associated with the translational apparatus. Corresponding to the overexpression of miR-520c-3p, knockdown of eIF4GII decreased protein levels of the miR-520c-3p target genes (Figure 5C). As illustrated in Figure 5D, examined mRNA levels were very low in non-translating and low-translating fractions of the gradient (fractions 1–7). In the Ctrl siRNA group, most of the transcripts were abundant in the actively translating polysomal fractions of the gradient (fractions 8–10). Silencing of eIF4GII led to a leftward shift in mRNA distribution, indicating that the studied transcripts associated with smaller polysomes and further suggesting that eIF4GII siRNA suppressed the initiation of mRNA translation.

As we observed, overexpression of pCDH-eIF4GII(CR) construct affected the expression of senescence markers (Figure 4E), therefore we wished to determine if eIF4GII repression might also participate in the process of miR-520c-3p triggered formation of the senescent phenotype. Comparable to the cells with increased miR-520c-3p, silencing of eIF4GII by siRNA caused a significant decline in the cell proliferation (Figure 5E) but no significant increase in cellular apoptosis (Figure 5F). Moreover, eIF4GII knockdown initiated cell cycle arrest in G_1_ phase (Figure 5G, Figure S6B), induced ß-galactosidase activity (Figure 5H) and altered levels of the senescence markers p16, p53, and HuR (Figure 5I). Collectively, these findings indicate that the miR-520c-3p-elicited repression in global translation is facilitated through the influence of miR-520c-3p on eIF4GII expression, and further eIF4GII actively contributes to the miR-520c-3p triggered decrease in cell proliferation and the induction of senescence.

### Both miR-520c-3p Overexpression and eIF4GII Knockdown Diminished the Clonogenic Capacity of Lymphoma Cells

**Figure 6:**
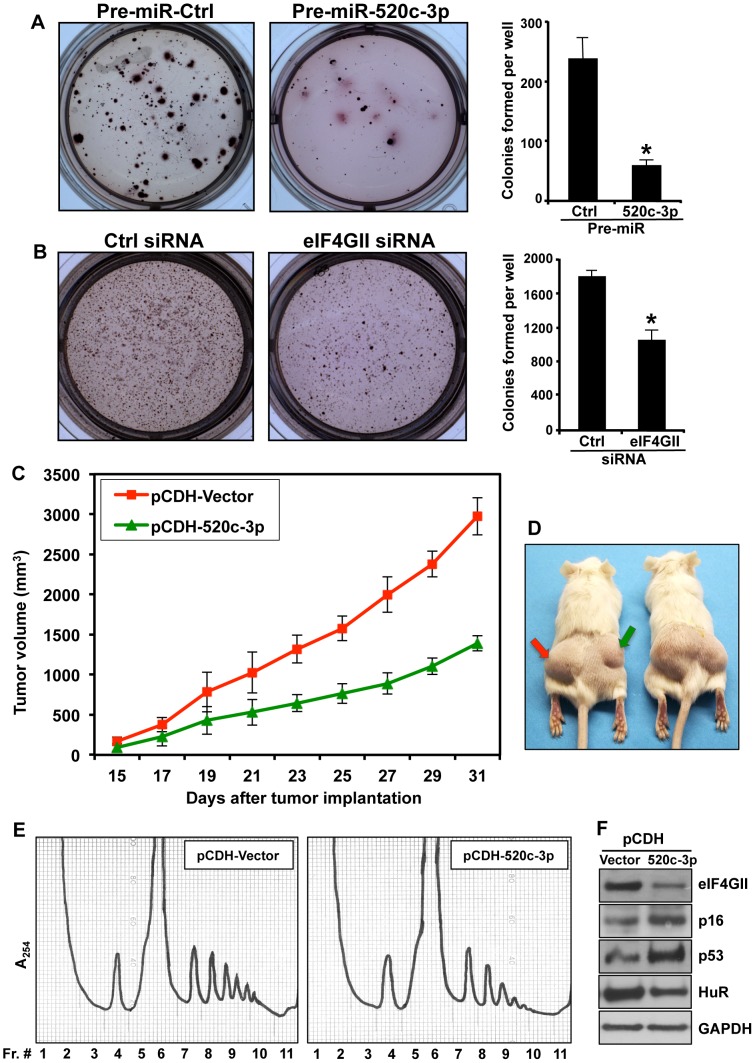
Overexpression of miR-520c-3p in DLBCL diminished colony formation in clonogenic assay and decreased tumor growth in xenograft model. (A) Farage cells after transfection with either Pre-miR-Ctrl or Pre-miR-520c-3p were cultured in agarose/medium for two weeks. Representative pictures (10×) are shown. Graphs represent the means and SD of three independent experiments. (B) Farage cells were transfected with Ctrl siRNA or eIF4GII siRNA, assay was performed and analyzed as described in (A). * p<0.05. (C and D) SCID Beige mice (n = 5) received a subcutaneous injection of SUDHL4 cells either expressing empty pCDH-Vector (on left sites; red arrow) or overexpressing miR-520c-3p, pCDH-520c-3p (on right sites; green arrow). The average tumor volume of each group with SEM is shown as a function of time. The repeated measure ANOVA showed a significant effect of time on tumors growth F(8,64) = 40.23, p<0.001, and significant repression of growth by miR-520c-3p as revealed by significant effect of treatment F(1,8) = 49.98, p<0.001 and significant treatment x time interaction F(8,64) = 5.93, p<0.001. (E) Lysates from SUDHL4 xenograft tumors obtained in C and D were fractionated by centrifugation through 10–50% linear sucrose gradients, and the polysome profiles were studied. (F) Protein extracts from xenograft tumors from (C) and (D) were subjected to Western blot analysis using indicated senescence markers antibodies.

Based on above data, we hypothesized that overexpression of miR-520c-3p can suppress tumor growth by decreasing protein translation, inhibiting proliferation, and promoting cell senescence. To address our hypothesis, we transfected Farage cells with either Pre-miRNA-Ctrl, Pre-miR-520c-3p (Figure 6A), siRNA targeting eIF4GII or control siRNA (Figure 6B) and carried out soft agar colony formation assays. As shown, either elevated miR-520c-3p levels or reduced eIF4GII levels significantly decreased the ability of cells to form colonies when compared to control transfections. These results provided further evidence that eIF4GII is implicated in miR-520c-3p mediated cells growth control.

### Increase in miR-520c-3p Expression Resulted in Repressed Tumor Formation in a Human Xenograft Model

Finally, we examined whether overexpression of miR-520c-3p inhibits tumor formation *in vivo*. To this end, we established a preclinical xenograft mouse model with SUDHL4 cells transduced with either an empty vector (pCDH-Vector) or a vector overexpressing miR-520c-3p (pCDH-520c-3p). Upregulation of miR-520c-3p resulted in a significant repression of lymphoma tumors growth (Figure 6C and D, Figure S7A). Consistently, similar results were obtained in a xenograft model performed with Pre-miR-520c-3p transfected HeLa cells (Figure S7B). To test if the tumors growth repression was influenced by translational inhibition, polysome distribution profiles were analyzed in tumors harvested from xenografts (Figures 6E). Indeed, such profiles were consistent with our *in vitro* cell culture data (Figure 1B and D) showing a global translational decrease in miR-520c-3p overexpressing tumors. Likewise, Western blot analysis of examined senescence markers in xenografts confirmed that the decrease in tumors growth was also affected by induced cellular senescence (Figure 6F). In summary, our findings provide strong evidence that miR-520c-3p overexpression contributes to the observed inhibition of tumor formation in the human xenograft mouse model by maintaining a translationally repressed state, inhibiting proliferation and initiating cellular senescence in cancer cells. We propose that the observed decreased protein translation and premature senescence is mediated, at least in part, through a miR-520c-3p-mediated decrease in expression of translational factor, eIF4GII.

### Reciprocal Expression of miR-520c-3p and eIF4GII in DLBCLs

**Figure 7:**
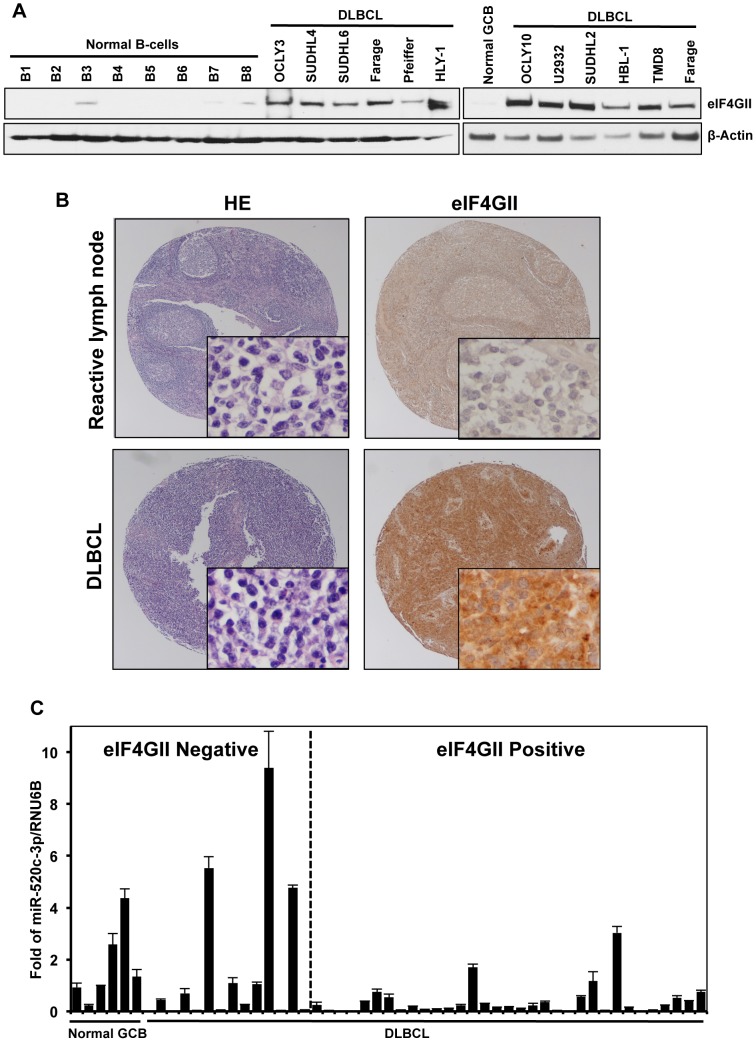
DLBCL cells express high levels of eIF4GII protein and low levels of miR-520c-3p. (A) eIF4GII protein levels in normal B-cells and DLBCL cells were analyzed by Western blot. β-Actin was used as a loading control. Data are representative of three independent experiments. (B) Pictured are representative fields (20× and 400× magnifications) of germinal center B-cell centroblasts of reactive lymph nodes and DLBCL tissue microarrays immunohistochemically stained with anti-eIF4GII antibody. HE staining was used for morphologic examination. (C) RNA from tissue microarrays used in (B) was purified and expression of miR-520c-3p was measured by RT-qPCR. Graphs represent the means and SEM from repeats of three independent assays.

Although eIF4GI has been found significantly elevated in a variety of cancers, to date, the tumorigenic potential of eIF4GII has not been investigated [16]–[19]. In the present study, we explored the expression of both miR-520c-3p levels and eIF4GII protein expression in a DLBCL model. As shown in Figure 1A, miR-520c-3p levels were consistently lower in both primary DLBCL cells and cultured DLBCL cell lines as compared to primary normal B-cells. Conversely, eIF4GII protein expression appeared to be low in normal B-cells and markedly elevated in all studied DLBCL cell lines (Figure 7A). To evaluate eIF4GII protein levels on primary DLBCL specimens and reactive lymph nodes, we performed immunohistochemistry analysis of tissue microarrays (TMA) on 49 primary DLBCL specimens (Table 1, Figure 7B). In agreement with Western blot analysis (Figure 7A), TMA data revealed that eIF4GII was markedly increased in 71% of all DLBCL (GCB and ABC subtype) compared to GCB of the reactive lymph nodes (14%) as summarized in Table 1 and representative sections shown in Figure 7B. Simultaneously, RNA from the same TMA samples was purified and expression of miR-520c-3p was measured by RT-qPCR (Table S3, Figure 7C). Despite the internal variability characteristic for primary samples derived from different patients we observed significant inverse relationship between eIF4GII protein expression and miR-520c-3p levels (Pearson correlation coefficient r = −0.38, p<0.02) in studied DLBCL cases. Altogether, this data support the role of miR-520c-3p and eIF4GII in the deregulation of protein synthesis in DLBCL and support the potential role of these molecules as specific therapeutic targets.

**Table 1:** Immunohistological analysis of eIF4GII expression in DLBCL and normal GCB.

Samples	*N*	Staining			% Positive
		Negative	Low	High	
DLBCL	49	14	24	11	71.43
GCB subtype	11	3	7	1	72.73
ABC subtype	38	11	17	10	71.05
Normal GCB	7	6	1	0	14.29

## Discussion

Numerous studies have established that miRNAs are incorporated in major critical cellular pathways and can function in concert with other genes crucial for control of cellular growth [20], [21]. Nevertheless, to understand the role of these small regulatory molecules in tumorigenesis, it is essential to identify the gene/pathway that they regulate. Herein, we demonstrated that elevated levels of miR-520c-3p resulted in inhibition of global gene translation, cell proliferation and promoted senescence in HeLa and DLBCL cells. Microarray analysis revealed that miR-520c-3p regulates the expression of many translation, cell proliferation and cancer related genes. Among those identified by microarray analysis and validated miR-520c-3p target genes, we found, eIF4GII, a pivotal scaffold member of eIF4F, and a central organizing protein in the recruitment of mRNA during translational initiation. Furthermore we observed that, comparable to miR-520c-3p elevation, silencing of eIF4GII by siRNA repressed translation, cell proliferation, promoted senescence and diminished the clonogenic ability of cancer cells. In addition, the overexpression of miR-520c-3p reduced eIF4GII translation and consequently decreased its protein levels through the miR-520c-3p seed sequence on the eIF4GII 3′UTR. Hence, by decreasing eIF4GII protein expression, miR-520c-3p indirectly controls expression of many eIF4GII target genes and collaboratively promotes changes in the proteome of malignant cells.

Multiple lines of evidence have now been established that the disruption of the translational machinery is a major contributor to cancer development and progression [8], [10]. Translational deregulation can occur through modification in the expression of proteins involved in translational initiation and/or changes in miRNA expression [33]–[35]. In recent years, a great deal of research has been focused on investigating the mechanisms of translational regulation by miRNAs. However, the role of miRNAs in control of specific factors of the translation initiation complex eIF4F has not yet been thoroughly explored. To date, only one report has described a miRNA dependent mechanism for the targeting of eIF4E and subsequent inhibition of host protein synthesis [38]. Thus, our results provide the first evidence of a miRNA regulating the translational initiation complex through the targeting of eIF4GII, and thereby modulating protein synthesis in cancer cells. Although, one cannot exclude the role of multiple target genes contributing to the observed miR-520c-3p triggered phenotype, signaling pathways (kinases or phosphatases) or other miRNAs actions that may also be involved, our data strongly support a major role of eIF4GII in this control.

While translational deregulation appears to be an essential feature of tumorigenesis and cellular senescence is considered to be tumor-suppressor mechanism there are few studies to date that have directly addressed the link between translational control and senescence. The results from these studies are confounding with some suggesting little difference in global rates of translation following oncogene-induced senescence [39], while others have shown that rapamycin treatment can actually prolong the induction of oncogene-induced senescence [40]. Some data have even suggested that the initiation of autophagy through inhibition of mTOR signaling (and presumably inhibition of translation) is a necessary prerequisite for senescence [41], [42]. It is thus of broad interest to determine what role translation or translational factors can play in the induction and maintenance of the cellular senescence program.

Consistent with our *in vitro* results, we demonstrated in a human xenograft mouse model that overexpression of miR-520c-3p significantly inhibits tumor formation *in vivo*. Although miR-520c has previously been linked to cancer [43]–[45], to date its role in tumor progression remains controversial and the molecular mechanism of miR-520c's function still awaits further clarification. We have identified the miR-520c-3p as a negative regulator of tumor growth via specific downregulation of translational factor, eIF4GII. Based on the data gathered from our studies, we postulate that the main tumor-suppressive mechanisms of miR-520c-3p can be attributed through the repression of translation and induction of premature senescence, which is a direct function of reduced eIF4GII protein levels. Recent reports indicate that mechanisms inducing premature senescence of cancer cells are emerging as a promising anti-tumorigenic, back-up program to improve on an insufficient apoptotic response [46], [47]. In addition, the combination of senescence induced therapy and a conventional therapy is postulated as a rational approach to cancer treatment [48], [49]. Accordingly, corresponding inhibition of translation and induction of senescence by miRNAs and/or small molecule inhibitors that target eIF4GII hold promise as an innovative strategy for cancer therapy.

MicroRNAs play a fundamental role in cancer biology and the alteration of their expression can have substantial effects on cell phenotypes. Thus, the role of miRNAs in the cancer pathogenesis makes them novel biomarkers and important therapeutic tools [50]. Accordingly, the results of several studies offer the experimental basis for the use of these small molecules in a therapeutic approach. For example, miR-34a and miR-155 have been already established as promising targets for lymphoma treatment [29]–[31]. While existing expression profiling studies have identified numerous miRNAs differentially expressed in lymphoma malignancies, DLBCL was found to have the most heterogeneous miRNA signature [26], [32], [51], [52]. Aberrant expression of miRNA can be a consequence of various mechanisms such as structural alterations, chromatin remodeling, aberrant transcription factor activity, etc. Encoded on chromosome 19q13.42, miR-520c was identified as being associated with t(4;14) and t(11;14) translocations in multiple myeloma. In addition, several other miRNAs located on chromosome 19q13.42, such as miR-520g, miR-520h, and miR-520b, have been found to be downregulated in multiple myeloma [53]. Although recurrent translocations involving chromosome 19q13 such as t(14;19)(q32.3;q13.2) have been reported in B-cell malignancies, they are extremely rare [54], [55]. Given that epigenetics has a profound effect on the regulation of miRNA expression and DNA hypermethylation has been shown to be involved in the decrease of miRNA in lymphomas, we examined if the decreased expression of miR-520c in DLBCLs as compared to normal B cells could be due to hypermethylation of the genomic locus (Text S1, Figure S8, Table S4). However, no hypermethylation within the studied locus was detected in lymphomas. We observed focal areas of hypomethylation of miR-520 and 5′ from the coding sequence in lymphomas, which may represent a permissive state for the binding of putative transcriptional repressors. Since specific miRNA can be deregulated by multiple mechanisms, examining the regulation of miR-520c repression in DLBCL by alternative means is an opportunity for future investigation.

We found that reduced miR-520c-3p was correlated with elevated eIF4GII expression in DLBCL. While the other functionally complementary scaffolding factors eIF4E and eIF4GI have been described as significantly increased in a variety of cancers [8]–[10], [17]–[19], our studies are the first to implicate eIF4GII in a malignancy. In the light of increasing evidence that abnormal function of cellular eIF4F complex caused by elevated expression of initiation factors plays a major role in tumorigenesis [56], it is widely believed that targeting the molecules of this complex is a rational cancer therapy [11], [34], [57]. Recently, promising research has been done on targeting eIF4E by using eIF4E-specific antisense nucleotides [4]. Also, small-molecule inhibitors have been utilized to block interactions between the translation initiation factors eIF4E and eIF4G1, and to modulate eIF4A activity [12], [13]. Our findings that eIF4GII is overexpressed in DLBCL provides an additional component of the eIF4F complex consideration as a potential therapeutic target.

In summary, we revealed a tumor-suppressive function of miR-520c-3p and identified eIF4GII as a major effector of this action. Our findings demonstrate the ability of miR-520c-3p to repress the development of cancer malignancies through coordinated control of several significant mechanisms: (1) inhibition of eIF4GII and consequent repression of global translational machinery and induction of senescence in tumor cells, and (2) corresponding regulation of other target genes involved in cancer development. Whereas much extant research has demonstrated the role of miRNA-target gene interactions for a single pathway, only a limited number of studies have shown the interaction of a specific miRNA with a major regulatory node, which has the potential to enhance pleiotropic phenotypic effects. The data presented herein not only illustrates the complexity of the miR-520c-3p triggered post-transcriptional regulation network that regulates translation and cell growth, but also provides information about the cooperation between the functionally related mechanisms that results in tumor growth repression. These *in vitro* and *in vivo* results were confirmed in DLBCL patient samples, where miR-520c-3p levels were inversely correlated to eIF4GII protein expression. Our study established a novel link between regulation of translation and senescence in malignant cells and suggests that protein synthesis control is a crucial part of the complex mechanism leading malignant cells to senescence program.

## Materials and Methods

### Ethics Statement

Human sample studies have been approved by the University of Maryland Medical School Institutional Review Board and conform to the Declaration of Helsinki.

Animal procedures were performed in compliance with protocols approved by the Institutional Animal Care and Use Committee of the University of Maryland.

### Cell Culture and Transfections

Human cervical carcinoma HeLa cells were cultured in DMEM (Gibco BRL, Gaithersburg, MD) supplemented with 10% FBS. Diffuse large B-cell lymphoma (DLBCL) and Burkitt lymphoma (BL) cell lines were cultured in RPMI medium 1640 (Gibco BRL, Gaithersburg, MD) containing 10% FBS. HeLa cells were transfected by using Oligofectamine (Invitrogen) with 50 nM small RNAs Pre-miR-520c-3p (AAAGUGCUUCCUUUUAGAGGGU), control Pre-miR-Ctrl (Ambion), or 20 nM siRNA targeting eIF4GII and control siRNA (Qiagen). Farage cells were transfected with Nucleofector Kit V (Amaxa, Inc., Germany). For transduction of SUDHL4 cells plasmids pCDH-Vector or pCDH-miR520c-3p (System Biosciences, CA) were transfected with PureFection (System Biosciences, CA) in HEK-293TN packaging cells. Afterward, SUDHL4 cells were co-cultivated with the viral particle producing HEK-293TN cells for 48 h, transferred to flasks and subjected to selection with 0.5 µg/ml puromycin. Plasmids pLenti-UTR-Luc-Blank or pLenti-UTR-Luc-eIF4GII(3′UTR) were purchased from Applied Biological Materials Inc. (BC, Canada), miRZip-Vector and miRZip-520c-3p constructs were from System Biosciences. Cloning of the eIF4GII 3′UTR fragments containing seed regions (wild-type or mutants) of miR-520c-3p in plasmid pcDNA3.1/NT-GFP-TOPO were performed using NT-GFP Fusion TOPO TA Expression Kit (Invitrogen). The EIF4G3 lentiviral expression vector was generated through amplification of EIF4G3's coding region (CD) from a human cDNA library and cloning the product into pCDH-EF1-MCS-IRES-Puro (Systems Bioscience).

### Primary Cells

Human sample studies have been approved by the University of Maryland Medical School Institutional Review Board and conform to the Declaration of Helsinki. Primary normal B cells were purified from human peripheral blood mononuclear cells (PBMCs) obtained from Sanguine Biosciences, Inc. (Valencia, CA). B cells isolation was performed with using human B Cell Isolation Kit (B-CLL) from Miltenyi Biotec Inc (Auburn, CA) according to manufacturer's protocol. Collected B cells purity was determined by flow cytometry. DLBCL primary cells were obtained from lymph node biopsies, followed by cell density ficoll-gradient separation. Tonsils were provided by the UMGCC Pathology Biorepository and Research Core to serve as normal Germinal Center B-cell (GCB) controls. GCBs were isolated from tonsils as previously described [58] with purity greater than 95%, CD19+, CD38+ and IgD-.

### Polyribosome Fractionation

Polysomal fractionations were carried out and microarray analysis was performed as previously described [59]. Briefly, cell lysates were fractionated by centrifugation through 10–50% linear sucrose gradients and divided into 11 fractions. RNA was purified from each fraction for microarray and RT-qPCR analysis.

### Microarray Data Analysis

RNA purified from sucrose fractions was labeled using Illumina TotalPrep RNA Amplification Kit (Ambion; Austin, TX). Human HT-12 v1.0 gene expression BeadChips containing 48,000 RefSeq transcripts (Illumina, San Diego, CA), were used for microarray analysis. Raw microarray data were filtered by the detection p-value≤0.02, normalized by Z-score transformation and tested for significant differences in signal intensity. The sample quality was analyzed by scatter plot, principal component analysis (PCA), and gene sample z-scores based hierarchy clustering to exclude possible outliers. ANOVA test was used to eliminate the genes with larger variances within each comparison group. Genes were considered to be significantly changed after calculating Z-ratio, indicating fold difference (Z>1.5 or <−1.5), false discovery rate (fdr), which controls for the expected proportion of false rejected hypothesis (fdr≤0.3) and p<0.05. Genes differentially expressed in studied groups were analyzed for representation of Gene Ontology (GO) terms to identify key biologically functional categories that were significantly changed (p<0.05) in miR-520c-3p overexpressed versus control transfected cells as described before [60]. Ingenuity Pathways Analysis IPA (https://analysis.ingenuity.com/; Ingenuity Systems; Redwood City, CA) was performed to identify the top network functions amongst the genes translationally regulated by miR-520c-3p. Network analysis utilizes a curated knowledge base on known functional interactions and protein functions to algorithmically infer biochemical interactions. Significance of functions and pathways was calculated using the right-tailed Fisher's Test. See www.ncbi.nlm.nih.gov/geo/query/acc.cgi?acc=GSE40489 for complete array results.

### RNA Analysis

Total cellular RNA or RNA from polysomal fractions were purified by using Trizol (Invitrogen), reverse transcribed with the iScript cDNA synthesis kit (BioRad, Hercules, CA) and quantified by real-time qPCR analysis using gene-specific primer pairs. qPCR analysis was carried out by using iQ SYBR Green Supermix (Quanta) and BioRad iCycler instrument. RNA from tissue microarrays (TMAs) was isolated using RecoverAll Total Nucleic Acid Isolation Kit for FFPE (Life Technologies) according to manufacturer's protocol. Mature miR-520c-3p and RNU6B were quantified using TaqMan MicroRNA Assays (Applied Biosystems). Each reaction was carried out in triplicate and three independent experiments were run.

### Oligonucleotides Used for Real Time RT-qPCR Analysis

For real-time qPCR quantification of selected miR-520c-3p targets following gene-specific primer pairs were used: CCCTTCAAGATGCACCTCAT and GTGGTCCACAGCATTTCCTT for eIF4GII mRNA, GATGCATTGCAGAGGCACTA and CCCTAATGCTGGAAGCACTC for FOXO3 mRNA, TGCAATCCAGCCAAATACAA and TTATCCAGCTTCAGCCAGGT for VIL2 mRNA, GGACCTTGGCCTCAGTGTTA and GAAAGTTGCAGGCACCAAAT for HNRPDL mRNA, TCCCCTTTCCCTGATCTTTT and TGGGATTCTGTGGGAGAAAG for HuR mRNA, TGCTATCATGGCAGAAGGAA and ATATGCCACAGCCCATCATT for MCT-1 mRNA and, CGGAGTCAACGGATTTGGTCGTAT and AGCCTTCTCCATGGTGGTGAAGAC for GAPDH mRNA.

### Analysis of Newly Translated Protein

For analysis of *de novo* translation, 72 h after transfection 10^6^ cells were incubated with 1 mCi L-[^35^S]methionine and L-[^35^S]cysteine (Easy Tag EXPRESS; PerkinElmer Life and Analytical Sciences, Waltham, MA) per 6-well plate for 20 minutes. Cell lysates were then resolved by SDS-PAGE and transferred onto PVDF membranes. Radiolabel incorporation was visualized by PhosphorImager (GE Healthcare).

### Western Blot Analysis

10–50 µg of whole cell lysates were resolved by SDS-PAGE (Invitrogen, Carlsbad, CA) and transferred onto PVDF membranes. After incubation with monoclonal antibodies recognizing eIF4GII (Sigma), p53, HuR, VIL2, GFP (all from Santa Cruz), GAPDH and β-Actin (both from Abcam, Cambridge, UK), or polyclonal FOXO3 (Cell Signaling), p16, HNRPDL, eIF4E (all from Santa Cruz), blots were incubated with the secondary antibodies (Santa Cruz). Signals were detected by enhanced chemiluminescence (Pierce, Rockford, IL).

### Assessment of Cell Cycle, Cell Proliferation, Cell Apoptosis and Senescence

72 h after transfections cells were fixed with 70% ethanol, washed with PBS and digested with RNAse (100 µm/ml) at 37 °C for 1 h. The cells were then stained with 50 µg/ml PI and analyzed for cell cycle with a flow cytometer. Cell proliferation in real-time was monitored by the xCELLigence System, RTCA DP Instrument (Roche Diagnostics GmbH, Germany). The cells were also stained with Trypan Blue (Sigma-Aldrich) and counted using a hemocytometer. The percentage of apoptotic cells was determined by flow cytometry with the Annexin V staining kit (BD Biosciences, CA). Senescence induction in the cells was tested by measurement of β-galactosidase activity with Senescence β-Galactosidase staining kit (Cell Signaling) and Western blot of p16, p53 and HuR senescence markers.

### m^7^GTP Pull-Down Assay

For cap-binding assay, 500 µg total cell lysates were incubated with a 7-Methyl GTP-Sepharose beads (GE Healthcare, Little Chalfont, UK) overnight at 4°C. After washing of the beads, the bound proteins were analyzed by Western blot.

### Colony Assay

Farage cells were transfected with Pre-miR-Ctrl, Pre-miR-520c-3p, eIF4GII siRNA or Ctrl siRNA and cultured in agarose/medium. 24 h later cells were plated at 7500 cells/ml in a 1∶3 agarose to DMEM mixture on the top of solidified 1∶1 agarose/DMEM feeder layer and covered with MDEM. Two weeks later colonies (>50 cells) were counted and pictures were taken with using Cell Colony Counter (Microbiology International).

### Tissue Microarrays (TMA) Staining and Immunohistologic Analysis

Tissue microarrays, containing 10 reactive lymph nodes, 54 DLBCL and 6 other types of lymphoma were purchased from Biomax and stained with Vectastain Elite ABC kit (Vector Laboratories) as described before [61]. Anti-eIF4GII antibody (Abcam) was used at 1∶1000 dilution, 4°C, overnight. Hematoxylin-eosin (HE) staining was used for morphologic examination. The DLBCL was classified into GCB and ABC subtypes, according to well-accepted immunohistochemistry-based algorithms [61]. The immunohistochemical staining was evaluated according to the following criteria: negative, <25% neoplastic cells staining; low, 26–50% neoplastic cells staining; high, >50% neoplastic cells staining. The images were analyzed with a Nikon eclipse 50i microscope (Nikon Instruments).

### *In Vivo* Tumor Growth in the Xenograft Models

Severe combined immunodeficiency (SCID) Beige mice were maintained in a pathogen–free environment under controlled conditions of light and humidity. Animal procedures were performed in compliance with protocols approved by the Institutional Animal Care and Use Committee of the University of Maryland. Six weeks old male mice were injected with 1.5×10^6^ SUDHL4 cells, either expressing empty vector or overexpressing miR-520c-3p into the left and right dorsal flanks, resuspended in 100 µL PBS and mixed with an equal volume of Matrigel. Tumors were measured and volumes were calculated as previously described [61], [62]. Thirty-one days after injection, tumors were harvested for further analysis. The effect of miR-520c-3p overexpression on tumor growth was evaluated using analysis of variance (ANOVA) with time as a repeated measure factor.

## Supporting Information

Figure S1 Overexpression of miR-520c-3p. (A) miR-520c-3p abundance as measured by RT-qPCR 72 h after transfection of HeLa cells with Pre-miR-Ctrl or Pre-miR-520c-3p. (B) 72 h after transfection as described in (A) HeLa cells were fractionated through sucrose gradients. Fractions containing ribosomal subunits 40S and 60S, monosomes 80S, and polysomes LMW and HMW (low- and high-molecular weight) are indicated. RNA was prepared from each fraction, and the relative intensity of the rRNA molecules is shown. (C) 72 h after transfection as described in (A) HeLa cells were harvested 20 min after incubation with ^35^S-labeled amino acids, size fractionated by SDS-PAGE, transferred onto PVDF membranes, and visualized using a PhosphorImager. Graph depicts quantification of the ^35^S-amino acid incorporation presented as percentage of signal intensity relative to control transfection. (D) miR-520c-3p levels were measured by RT-qPCR 72 h after transduction of SUDHL4 cells with either empty vector (pCDH-Vector) or vector overexpressing miR-520c-3p (pCDH-520c-3p), or transfecting Farage cells with Pre-miR-Ctrl or Pre-miR-520c-3p. (E) SUDHL4 cells were transducted as described in (D) and analyzed 72 h later as described in (B). (F) Cells were transducted as described in (D) and analyzed 72 h later as described in (C).
(TIFF)

Figure S2 Downregulation of miR-520c-3p. (A and B) 48 h after transduction with miRZip-Vector or miRZip-520c-3p HeLa cells were analyzed for global protein synthesis as described in Figure S1B and C, respectively. (C and D) SUDHL4 cells were transducted and analyzed as described in (A and B).
(TIFF)

Figure S3 Cell cycle analysis. (A) Cells were stained with PI and subjected to cell cycle analysis 72 h after transfection with Pre-miR-Ctrl or Pre-miR-520c-3p (HeLa) or transduction with pCDH-Vector or pCDH-520c-3p (SUDHL6). (B) Cells were transfected/transducted as described in (A) and cell cycle was analyzed 48 h later. Representative pictures are shown. Graphs represent the means and SEM from three repeats of three independent assays.
(TIFF)

Figure S4 Top 100 functional annotations for total and polysome associated mRNAs in Pre-miR-520c-3p compared to Pre-miR-Ctrl transfected HeLa cells identified by GO analysis. T represents total RNA; lanes 1 through 11 represent RNA from sucrose fractions of increasing molecular weight.
(TIFF)

Figure S5 Half-lives of validated in Figure 3 mRNAs were measured by incubating cells with actinomycin D (4 µg/ml), extracting total RNA at the indicated times, and measuring mRNAs normalized to GAPDH mRNA by RT-qPCR analysis.
(TIFF)

Figure S6 (A) (Upper) Schematic of GFP reporter constructs bearing segments with predicted miR-520c-3p sites with either wild type (wt) or with mutated (mut) seed sequences on eIF4GII 3′UTR. (Lower) GFP mRNA levels measured by RT-qPCR 48 h after cotransfection of above plasmids with Pre-miR-Ctrl or Pre-miR-520c-3p. (B) 72 h after transfection with Ctrl siRNA or eIF4GII siRNA, HeLa cells were stained with PI and subjected to cell cycle analysis. Representative pictures are shown.
(TIFF)

Figure S7 Xenograft tumors in SCID mice using cells overexpressing miR-520c-3p. (A) mRNAs extracted from SUDHL4 xenograft tumors were subjected to RT-qPCR for validation of miR-520c-3p levels. (B) Mice (n = 6) received a subcutaneous injection of HeLa cells either transfected with Pre-miR-Ctrl or Pre-miR-520c-3p. Tumors were measured and volumes were calculated as described in the paper. The repeated measure ANOVA showed a significant effect of time on tumors growth F(13,78) = 10.05, p<0.001, and significant inhibition of growth by miR-520c-3p as revealed by significant effect of treatment F(1,7) = 40.60, p<0.001 and significant treatment x time interaction F(13,78) = 2.04, p<0.05.
(TIFF)

Figure S8 Interrogation of a 2 kB locus around miR520C using Mass Array Sequenom Epityping reveals focal losses of methylation, but no gains of methylation in DLBCL cell lines and primary cases, as compared to normal GCB cells. Columns correspond to each interrogated CpG within the amplicon (CpGs in columns correspond to the indicated genomic location as visualized in UCSC browser and reflect methylation either within the coding sequence of the gene or adjacent 5′ and 3′ sequences). We profiled 4 fractions of isolated GCBs, 6 DLBCL cell lines and 9 primary DLBCL cases.
(TIFF)

Table S1 The top five functional networks derived by Ingenuity Pathways Analysis (IPA) from the genes translationally regulated by miR-520c-3p.
(DOC)

Table S2 List of genes with the most significantly altered Z-ratio in Pre-miR-520c-3p compared to Pre-miR-Ctrl transfected HeLa cells.
(DOC)

Table S3 eIF4GII (IHC staining) and miR-520c-3p (measured by RT-qPCR) expression in TMA of primary DLBCL samples. 0 - negative staining, 1 - positive staining.
(DOCX)

Table S4 EpiTYPER primer sequences designed for miR520c.
(DOC)

Text S1 Single Locus Quantitative DNA Methylation Assays.
(DOC)
