# Supplementary figures and images for: Down-Regulation of eIF4GII by miR-520c-3p Represses Diffuse Large B Cell Lymphoma Development
Source: PLoS Genet. 2014 Jan 30;10(1):e1004105. doi: 10.1371/journal.pgen.1004105 (PMC3907297; doi:10.1371/journal.pgen.1004105)

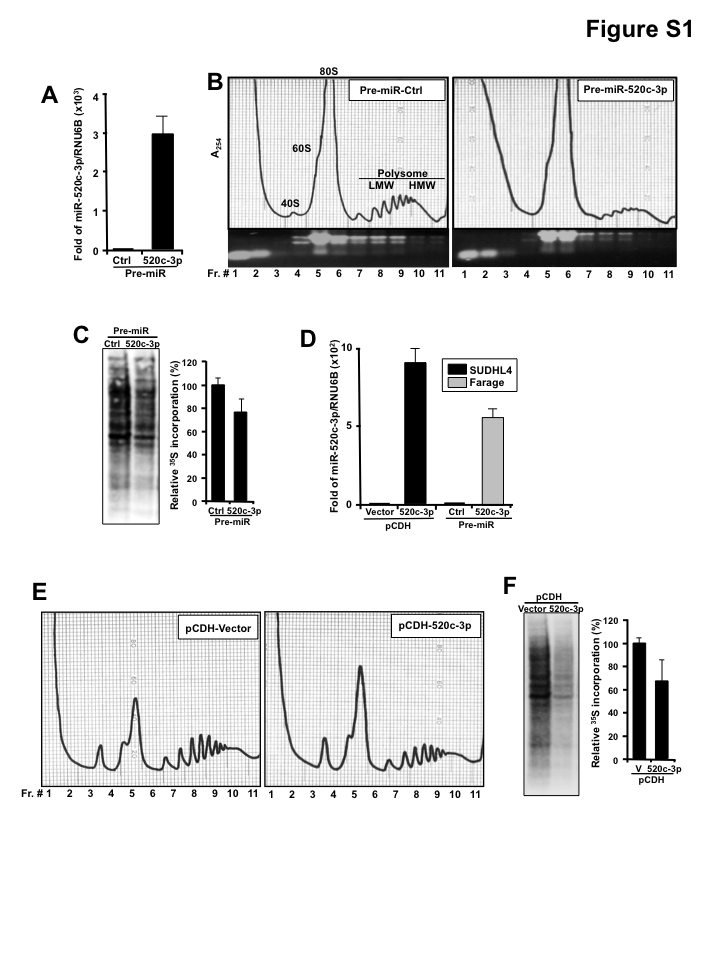

Supplement: Figure S1 — Overexpression of miR-520c-3p. (A) miR-520c-3p abundance as measured by RT-qPCR 72 h after transfection of HeLa cells with Pre-miR-Ctrl or Pre-miR-520c-3p. (B) 72 h after transfection as described in (A) HeLa cells were fractionated through sucrose gradients. Fractions containing ribosomal subunits 40S and 60S, monosomes 80S, and polysomes LMW and HMW (low- and high-molecular weight) are indicated. RNA was prepared from each fraction, and the relative intensity of the rRNA molecules is shown. (C) 72 h after transfection as described in (A) HeLa cells were harvested 20 min after incubation with 35S-labeled amino acids, size fractionated by SDS-PAGE, transferred onto PVDF membranes, and visualized using a PhosphorImager. Graph depicts quantification of the 35S-amino acid incorporation presented as percentage of signal intensity relative to control transfection. (D) miR-520c-3p levels were measured by RT-qPCR 72 h after transduction of SUDHL4 cells with either empty vector (pCDH-Vector) or vector overexpressing miR-520c-3p (pCDH-520c-3p), or transfecting Farage cells with Pre-miR-Ctrl or Pre-miR-520c-3p. (E) SUDHL4 cells were transducted as described in (D) and analyzed 72 h later as described in (B). (F) Cells were transducted as described in (D) and analyzed 72 h later as described in (C). (TIFF) [file pgen.1004105.s001.tiff]

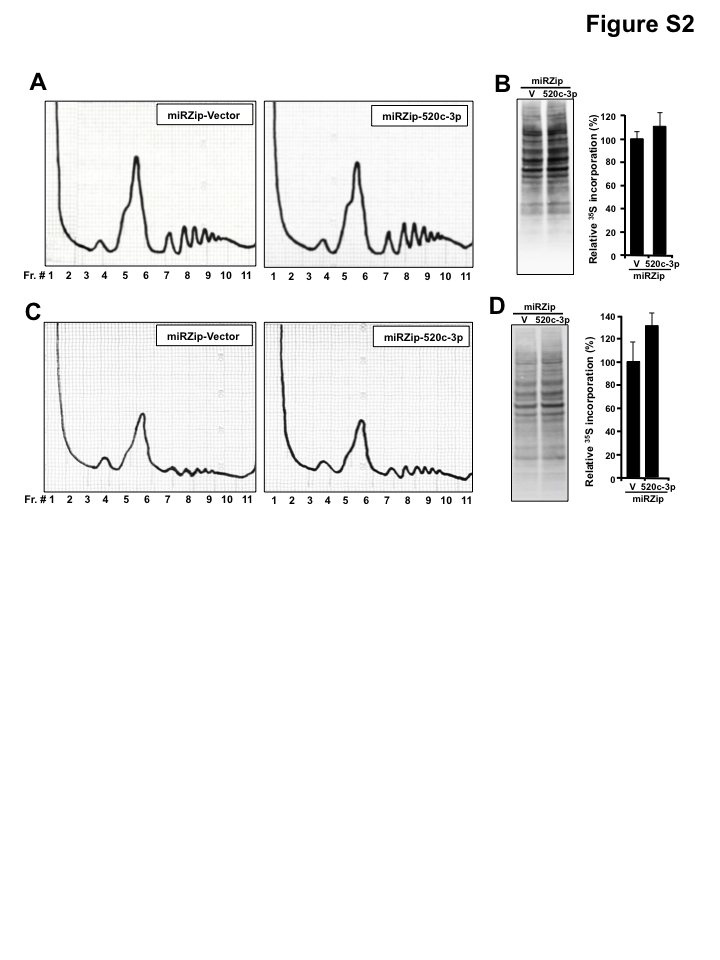

Supplement: Figure S2 — Downregulation of miR-520c-3p. (A and B) 48 h after transduction with miRZip-Vector or miRZip-520c-3p HeLa cells were analyzed for global protein synthesis as described in Figure S1B and C, respectively. (C and D) SUDHL4 cells were transducted and analyzed as described in (A and B). (TIFF) [file pgen.1004105.s002.tiff]

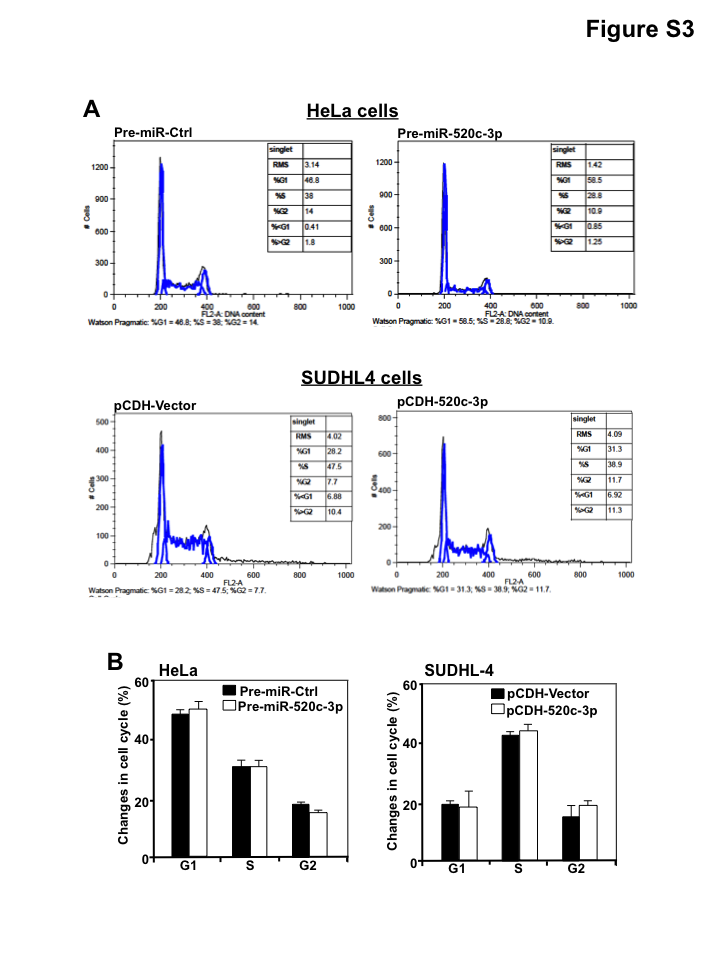

Supplement: Figure S3 — Cell cycle analysis. (A) Cells were stained with PI and subjected to cell cycle analysis 72 h after transfection with Pre-miR-Ctrl or Pre-miR-520c-3p (HeLa) or transduction with pCDH-Vector or pCDH-520c-3p (SUDHL6). (B) Cells were transfected/transducted as described in (A) and cell cycle was analyzed 48 h later. Representative pictures are shown. Graphs represent the means and SEM from three repeats of three independent assays. (TIFF) [file pgen.1004105.s003.tiff]

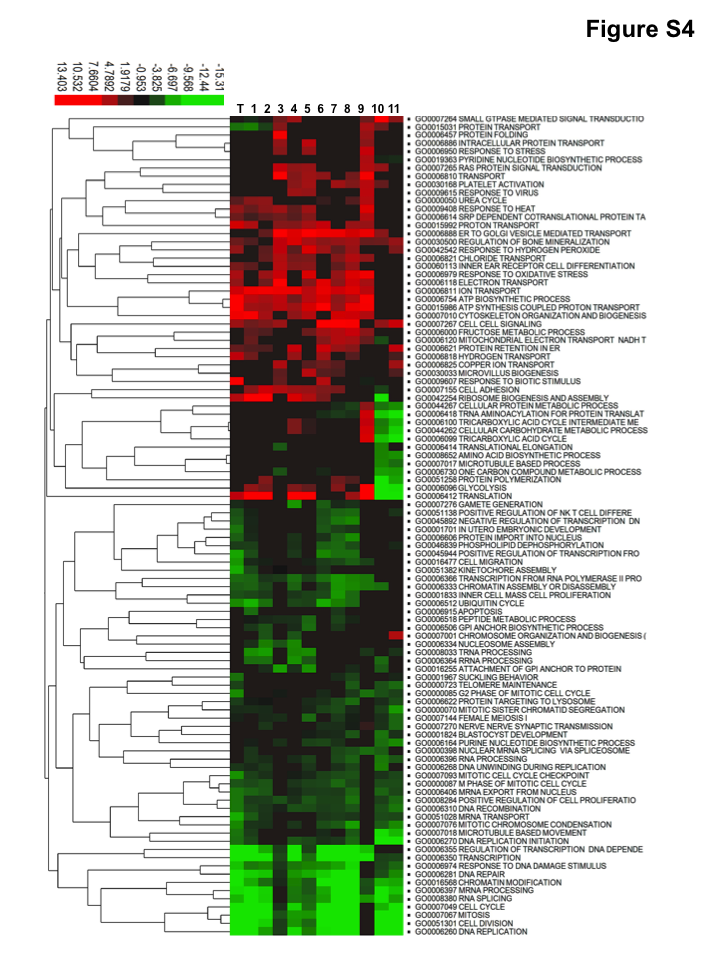

Supplement: Figure S4 — Top 100 functional annotations for total and polysome associated mRNAs in Pre-miR-520c-3p compared to Pre-miR-Ctrl transfected HeLa cells identified by GO analysis. T represents total RNA; lanes 1 through 11 represent RNA from sucrose fractions of increasing molecular weight. (TIFF) [file pgen.1004105.s004.tiff]

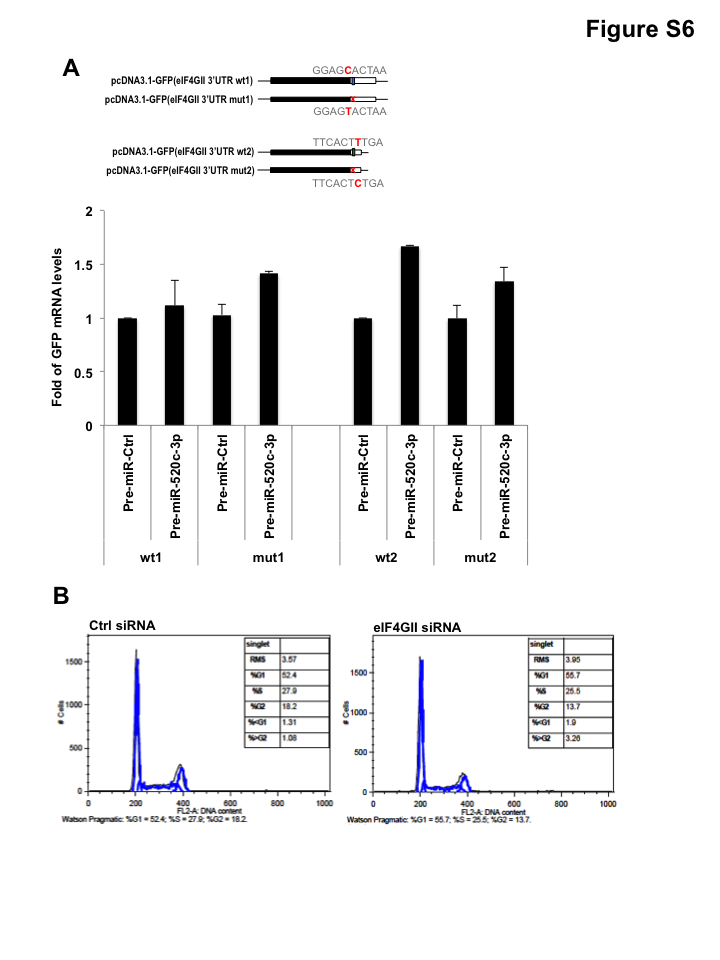

Supplement: Figure S6 — (A) (Upper) Schematic of GFP reporter constructs bearing segments with predicted miR-520c-3p sites with either wild type (wt) or with mutated (mut) seed sequences on eIF4GII 3′UTR. (Lower) GFP mRNA levels measured by RT-qPCR 48 h after cotransfection of above plasmids with Pre-miR-Ctrl or Pre-miR-520c-3p. (B) 72 h after transfection with Ctrl siRNA or eIF4GII siRNA, HeLa cells were stained with PI and subjected to cell cycle analysis. Representative pictures are shown. (TIFF) [file pgen.1004105.s006.tiff]

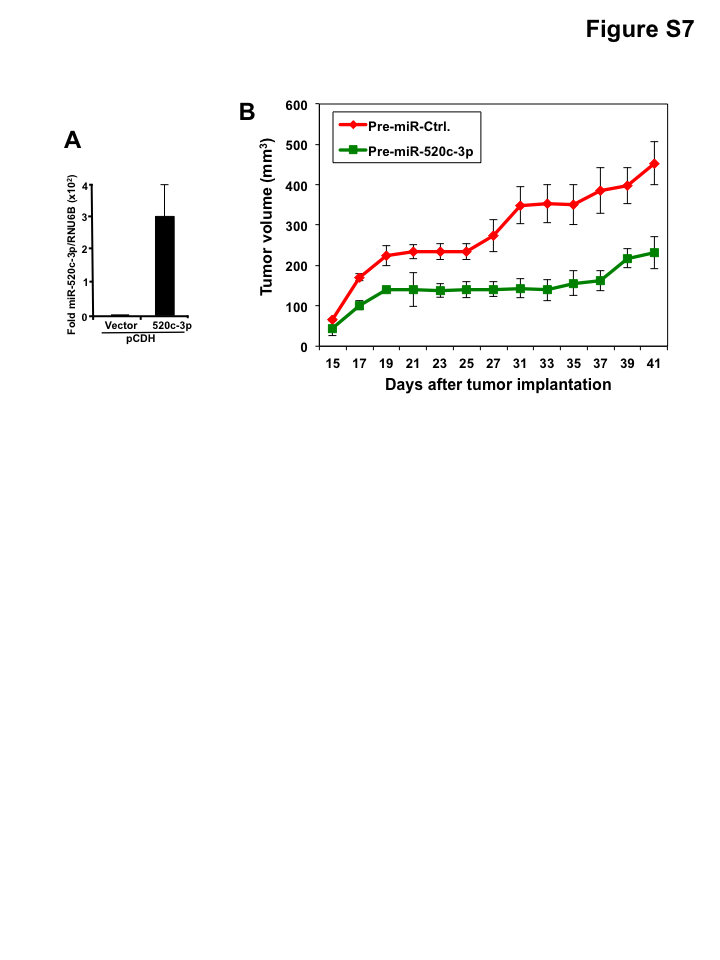

Supplement: Figure S7 — Xenograft tumors in SCID mice using cells overexpressing miR-520c-3p. (A) mRNAs extracted from SUDHL4 xenograft tumors were subjected to RT-qPCR for validation of miR-520c-3p levels. (B) Mice (n = 6) received a subcutaneous injection of HeLa cells either transfected with Pre-miR-Ctrl or Pre-miR-520c-3p. Tumors were measured and volumes were calculated as described in the paper. The repeated measure ANOVA showed a significant effect of time on tumors growth F(13,78) = 10.05, p<0.001, and significant inhibition of growth by miR-520c-3p as revealed by significant effect of treatment F(1,7) = 40.60, p<0.001 and significant treatment x time interaction F(13,78) = 2.04, p<0.05. (TIFF) [file pgen.1004105.s007.tiff]

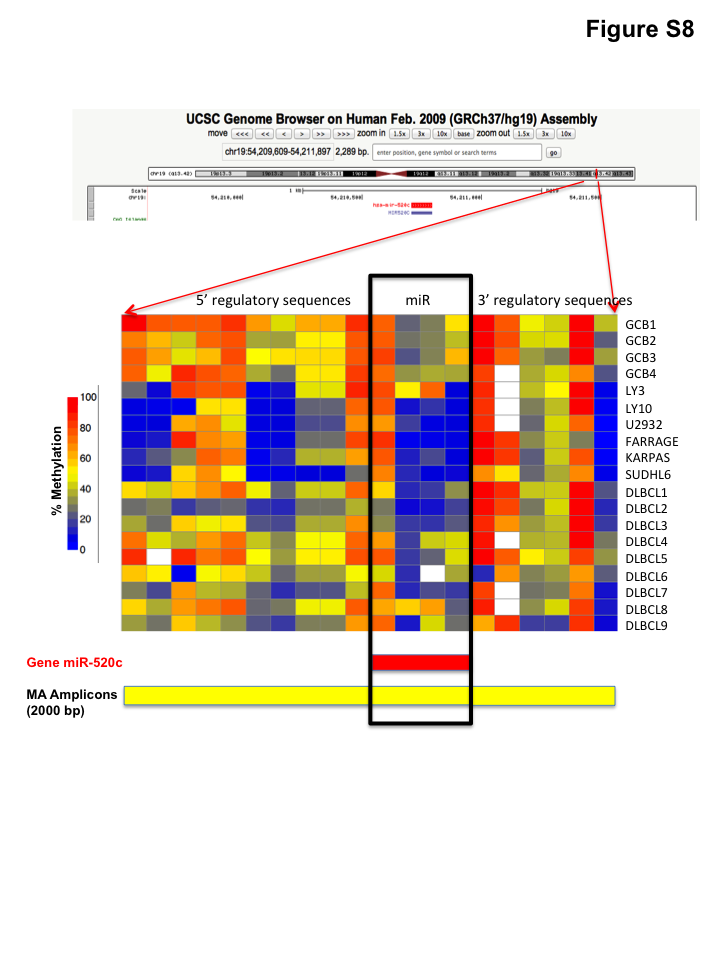

Supplement: Figure S8 — Interrogation of a 2 kB locus around miR520C using Mass Array Sequenom Epityping reveals focal losses of methylation, but no gains of methylation in DLBCL cell lines and primary cases, as compared to normal GCB cells. Columns correspond to each interrogated CpG within the amplicon (CpGs in columns correspond to the indicated genomic location as visualized in UCSC browser and reflect methylation either within the coding sequence of the gene or adjacent 5′ and 3′ sequences). We profiled 4 fractions of isolated GCBs, 6 DLBCL cell lines and 9 primary DLBCL cases. (TIFF) [file pgen.1004105.s008.tiff]
