# Supplementary material for: Down-Regulation of eIF4GII by miR-520c-3p Represses Diffuse Large B Cell Lymphoma Development
Source: PLoS Genet. 2014 Jan 30;10(1):e1004105. doi: 10.1371/journal.pgen.1004105 (PMC3907297; doi:10.1371/journal.pgen.1004105)
Supplement: Table S1 — The top five functional networks derived by Ingenuity Pathways Analysis (IPA) from the genes translationally regulated by miR-520c-3p. (DOC) [file pgen.1004105.s009.doc]

**Table S1**

| **ID** | **Associated Network Functions** | **Score** |
| --- | --- | --- |
| 1 | RNA Post-Transcriptional Modification, Cellular Development, Cellular Growth and Proliferation | 44 |
| 2 | Molecular Transport, Antigen Presentation, Cell Cycle | 44 |
| 3 | Cancer, Hematological Disease, Nucleic Acid Metabolism | 40 |
| 4 | Cellular Assembly and Organization, Cellular Function and Maintenance, Genetic Disorder | 40 |
| 5 | Nucleic Acid Metabolism, Small Molecule Biochemistry, Cell-To-Cell Signaling and Interaction | 39 |
