# Supplementary material for: Down-Regulation of eIF4GII by miR-520c-3p Represses Diffuse Large B Cell Lymphoma Development
Source: PLoS Genet. 2014 Jan 30;10(1):e1004105. doi: 10.1371/journal.pgen.1004105 (PMC3907297; doi:10.1371/journal.pgen.1004105)
Supplement: Table S2 — List of genes with the most significantly altered Z-ratio in Pre-miR-520c-3p compared to Pre-miR-Ctrl transfected HeLa cells. (DOC) [file pgen.1004105.s010.doc]

Table S2

| **Entrez**  **Gene ID** | **Gene**  **Name** | **Z-ratio** | | | | | | | | | | | | **p-value** | | | | | | | | | | | |
| --- | --- | --- | --- | --- | --- | --- | --- | --- | --- | --- | --- | --- | --- | --- | --- | --- | --- | --- | --- | --- | --- | --- | --- | --- | --- |
|  |  | **Fr.1** | **Fr.2** | **Fr.3** | **Fr.4** | **Fr.5** | **Fr.6** | **Fr.7** | **Fr.8** | **Fr.9** | **Fr.10** | **Fr.11** | **Total** | **Fr.1** | **Fr.2** | **Fr.3** | **Fr.4** | **Fr.5** | **Fr.6** | **Fr.7** | **Fr.8** | **Fr.9** | **Fr.10** | **Fr.11** | **Total** |
| 26574 | AATF | -0.67 | -0.52 | 0.52 | -1.54 | -1.09 | -1.2 | -0.7 | -2.85 | -1.52 | -2.1 | -1.14 | -1.28 | 0.2517 | 0.5572 | 0.6954 | 0.0359 | 0.0861 | 0.161 | 0.4165 | 0.0273 | 0.0067 | 0.0542 | 0.6106 | 0 |
| 10120 | ACTR1B | -2.28 | -1.96 | -1.81 | -2.5 | -4.4 | -1.36 | -3 | -2.79 | -4.42 | 1.28 | 1.22 | -0.84 | 0.0004 | 0.0017 | 0.0001 | 0.1247 | 0.003 | 0.0504 | 0 | 0 | 0.0001 | 0.4041 | 0.0045 | 0.2673 |
| 132 | ADK | 0.08 | 0.81 | 1.08 | 0.3 | 1.78 | 1.64 | 2.07 | 2.1 | 1.6 | 0.85 | 0.38 | 0.31 | 0.9004 | 0.2873 | 0.0014 | 0.7523 | 0.1501 | 0.0585 | 0.0173 | 0.0052 | 0.0008 | 0.1834 | 0.6521 | 0.862 |
| 64400 | AKTIP | -2.54 | -2.67 | -2.62 | -1.55 | -3.38 | -4.13 | -3.34 | -1.9 | -0.79 | 2.16 | -0.67 | -2.57 | 0 | 0.0075 | 0.0016 | 0.1098 | 0.1153 | 0.0013 | 0.0203 | 0.0037 | 0.3513 | 0.1899 | 0.7609 | 0.0004 |
| 10541 | ANP32B | -0.17 | -1.83 | -1.08 | -0.49 | 0.07 | -1.76 | -1.41 | -1.5 | -1.94 | -0.32 | 1.29 | 0.07 | 0.9105 | 0.0166 | 0.6736 | 0.5928 | 0.8592 | 0.0312 | 0.0061 | 0.3284 | 0.3093 | 0.744 | 0.4453 | 0.9799 |
| 328 | APEX1 | -1.21 | -4.36 | -3.84 | -0.63 | -2.26 | -2.59 | -3.21 | -2.32 | -1.38 | -0.98 | -2.65 | -1.44 | 0.1868 | 0.0007 | 0.1556 | 0.7165 | 0.1249 | 0.0005 | 0.0096 | 0.0009 | 0.0075 | 0.0222 | 0.0396 | 0.3844 |
| 10093 | ARPC4 | -0.04 | 1.55 | 0.26 | 0.47 | 2.11 | 4.25 | 2.59 | 2.51 | 0.95 | 0.74 | 5.71 | 1.61 | 0.8759 | 0.0791 | 0.8749 | 0.6136 | 0.0034 | 0.0004 | 0 | 0.0359 | 0.3881 | 0.3391 | 0.0042 | 0.5062 |
| 84126 | ATRIP | -1.59 | -2.02 | -1.92 | -2.06 | -3.49 | -2.34 | -4.47 | -2.47 | -2.88 | -1.18 | -0.59 | -0.3 | 0.0673 | 0.0755 | 0.0222 | 0.1958 | 0.0012 | 0.0065 | 0 | 0.0002 | 0.0001 | 0.0802 | 0.3231 | 0.8011 |
| 84126 | ATRIP | -1.1 | -2.48 | -1.76 | -1.45 | -2.31 | -1.85 | -3 | -3.1 | -2.42 | -0.31 | -0.06 | -0.23 | 0.1093 | 0.0011 | 0.1035 | 0.134 | 0.0219 | 0.0524 | 0.0117 | 0.0014 | 0.0001 | 0.6686 | 0.938 | 0.7516 |
| 9334 | B4GALT5 | -2.11 | -1.95 | 0.03 | -2.09 | -1.18 | -3.17 | -3.71 | -2.98 | -2.07 | 2.89 | 2.14 | -1.47 | 0.0001 | 0.2592 | 0.9554 | 0.4242 | 0.669 | 0.1606 | 0.028 | 0.004 | 0 | 0.1727 | 0.3701 | 0.3797 |
| 9275 | BCL7B | -2.83 | -2.15 | -0.07 | 0.12 | -2.7 | -1.65 | -2.11 | -3.13 | -2.79 | 1.83 | 0.82 | -1.64 | 0 | 0.0148 | 0.9766 | 0.9661 | 0.0053 | 0.022 | 0.086 | 0.0005 | 0 | 0.4106 | 0.4428 | 0 |
| 2647 | BLOC1S1 | -1.65 | -2.07 | -1.32 | -3.39 | -2.64 | 0.5 | -3.26 | -2.32 | -4.82 | 2.17 | 3.1 | 1.7 | 0.3208 | 0.2743 | 0.0096 | 0.2143 | 0.0146 | 0.4966 | 0.0002 | 0.0858 | 0.0027 | 0.0674 | 0.0089 | 0.0003 |
| 388962 | BOLA3 | 1.12 | -0.87 | 1.34 | 0.01 | -0.63 | -1.94 | -2.49 | -0.29 | -3.08 | 3.51 | 2.49 | 0.01 | 0.5799 | 0.5126 | 0.6202 | 0.9971 | 0.1746 | 0.0152 | 0.0011 | 0.8053 | 0.0023 | 0.3097 | 0.3316 | 0.9912 |
| 672 | BRCA1 | -2.38 | -2.48 | -0.56 | -1.68 | -0.68 | -1.8 | -1.81 | -2.92 | -3.28 | -0.75 | -0.12 | -2.6 | 0.0078 | 0.0009 | 0.3089 | 0.035 | 0.4529 | 0.0022 | 0.0125 | 0.0034 | 0.0152 | 0.6037 | 0.7183 | 0 |
| 8019 | BRD3 | -2.94 | 0.19 | -0.52 | -2.22 | -0.82 | -3.76 | -4.21 | -4.14 | -1.95 | -2.05 | 5.21 | -2.05 | 0 | 0.8696 | 0.3884 | 0.0743 | 0.6864 | 0.0186 | 0.017 | 0 | 0.3842 | 0.483 | 0.0002 | 0.231 |
| 725 | C4BPB | -2.4 | -2.11 | -0.85 | -2.93 | -3.3 | -3.18 | -2.66 | -0.96 | -2.2 | -0.24 | -0.97 | -1.37 | 0 | 0.0396 | 0.5102 | 0.1186 | 0 | 0.0028 | 0.0007 | 0.2553 | 0.0957 | 0.734 | 0.399 | 0.3636 |
| 726 | CAPN5 | 1.18 | 1.17 | 0.61 | 1.39 | 1.36 | 1.8 | 1.03 | 2.77 | 2.38 | 1.18 | 1.81 | 1.35 | 0 | 0.0003 | 0.0051 | 0 | 0 | 0.0001 | 0.4973 | 0 | 0 | 0.0061 | 0.104 | 0 |
| 114769 | COP1 | -0.13 | 1.04 | 2.57 | 1.84 | 3.76 | 2.96 | 1.21 | 3.32 | 2.07 | 2.44 | 2.75 | 2.51 | 0.8968 | 0.248 | 0.0001 | 0.2057 | 0 | 0 | 0.4597 | 0 | 0.0035 | 0 | 0.0009 | 0 |
| 840 | CASP7 | 0 | 0.45 | -0.11 | -0.17 | -1.13 | -0.75 | -0.39 | -1.31 | 0.14 | -0.86 | -0.88 | -0.11 | 0.9967 | 0.0807 | 0.5078 | 0.6272 | 0 | 0.0167 | 0.3026 | 0.0022 | 0.781 | 0.0042 | 0.2533 | 0.9341 |
| 840 | CASP7 | -0.97 | -0.67 | -0.31 | -0.24 | -1.23 | -1.32 | -3.19 | -2.27 | -0.63 | -1.35 | -2.77 | -1.19 | 0.0989 | 0 | 0.7187 | 0.6092 | 0.0451 | 0.0026 | 0.0009 | 0 | 0 | 0.0063 | 0.0807 | 0.4359 |
| 842 | CASP9 | 2.72 | 5.04 | 5.91 | 4.2 | 4.86 | 4.94 | 4.65 | 5.34 | 7.68 | 2.8 | 2.35 | 2.95 | 0.0265 | 0 | 0.0054 | 0.0018 | 0 | 0 | 0 | 0 | 0 | 0.2009 | 0.3436 | 0 |
| 9133 | CCNB2 | -4.36 | -4.27 | -4.46 | -3.05 | -3.2 | -2.91 | -3.99 | -2.49 | -1.62 | -2.03 | -2.64 | -1.92 | 0 | 0 | 0.0008 | 0.0023 | 0.0771 | 0.0052 | 0.011 | 0 | 0.0124 | 0 | 0.0017 | 0 |
| 994 | CDC25B | -1.78 | -2.05 | -2.06 | -2.43 | -3.34 | -1.68 | -2.99 | -1.78 | -0.85 | -2.57 | -3.52 | -0.42 | 0 | 0.015 | 0 | 0.1404 | 0.0022 | 0.0001 | 0 | 0.0483 | 0.4041 | 0.1101 | 0.009 | 0.436 |
| 995 | CDC25C | -0.9 | -0.92 | -0.32 | -0.08 | -1.83 | -1.72 | -1.68 | -1.92 | -0.63 | -1.42 | -1.05 | -1.42 | 0.0021 | 0.0793 | 0.0243 | 0.6739 | 0 | 0 | 0.0051 | 0.0149 | 0.1788 | 0.0101 | 0.086 | 0 |
| 984 | CDC2L1 | -0.49 | 0.39 | 1.05 | 0.49 | 0.55 | -0.48 | -0.18 | -3.17 | -2.78 | -1.3 | -0.12 | -1.23 | 0.2475 | 0.2434 | 0 | 0.5296 | 0.0792 | 0 | 0.6259 | 0 | 0 | 0.0024 | 0.923 | 0.0033 |
| 728642 | CDC2L2 | -0.67 | 0.52 | 0.3 | 0.49 | -0.12 | 0.44 | 0.07 | -0.65 | -2.76 | -1.08 | 0.15 | -0.29 | 0.4422 | 0.1231 | 0.0076 | 0.5867 | 0.7201 | 0.1875 | 0.8164 | 0.1249 | 0.0306 | 0.0483 | 0.2962 | 0.6135 |
| 10435 | CDC42EP2 | -0.31 | -0.08 | 2.13 | 2.57 | 2.03 | 3.16 | 3.03 | 3.54 | -0.26 | 2.67 | 3.46 | 0.96 | 0.643 | 0.8837 | 0.4842 | 0.2836 | 0.3155 | 0.0007 | 0.0352 | 0 | 0.8396 | 0.0138 | 0 | 0.5503 |
| 56990 | CDC42SE2 | -0.49 | 0.02 | 2.87 | 4.2 | 3.49 | 0.82 | 1.55 | 2.37 | 3.03 | 4.5 | 1.78 | -0.42 | 0.4103 | 0.9886 | 0.1444 | 0.05 | 0.2319 | 0.6082 | 0.5565 | 0.001 | 0.0272 | 0.0687 | 0.5696 | 0.8516 |
| 83461 | CDCA3 | -3.47 | -4.82 | -2.39 | -3.18 | -5.81 | -2.1 | -4.99 | -3.36 | -4.55 | -1.14 | -2.23 | -2.19 | 0 | 0 | 0.2681 | 0.0303 | 0.0011 | 0.0436 | 0.0008 | 0.0045 | 0 | 0.0128 | 0 | 0.3167 |
| 55143 | CDCA8 | -3.45 | -2.43 | -2.87 | -1.86 | -1.6 | -0.83 | -3.93 | -3.23 | -2.52 | -2.96 | -2.93 | -2.58 | 0 | 0 | 0.0455 | 0.0666 | 0.0002 | 0.1139 | 0 | 0 | 0.0001 | 0.0794 | 0.0602 | 0.0361 |
| 1029 | CDKN2A | -2.01 | -3.19 | -0.9 | -2.37 | -3.54 | 1.16 | -2.65 | -1.63 | -3.86 | 2.1 | 4.86 | -0.25 | 0 | 0.0317 | 0.6064 | 0.1995 | 0.0001 | 0.2617 | 0.0095 | 0.0057 | 0.0106 | 0.2639 | 0.0536 | 0.9403 |
| 79019 | CENPM | -3.06 | -3.36 | -2.9 | -3.13 | -4.83 | -0.42 | -2.95 | -2.42 | -3.56 | -1.46 | 2.61 | -0.79 | 0 | 0 | 0.1449 | 0.033 | 0 | 0.5136 | 0 | 0.0174 | 0.0108 | 0.0389 | 0.3168 | 0.8363 |
| 79019 | CENPM | -3.6 | -3.42 | -2.5 | -4.38 | -4.28 | -1.9 | -2.26 | -2.05 | -3.74 | -1.79 | 1.12 | -1.23 | 0 | 0.0014 | 0.1537 | 0.0621 | 0 | 0.0005 | 0.0059 | 0.1079 | 0.0014 | 0.1003 | 0.4557 | 0.7194 |
| 79019 | CENPM | -3.82 | -3.49 | -3.29 | -4.88 | -5.45 | -1.32 | -2.45 | -1.04 | -4.16 | -1.51 | -0.83 | 0.47 | 0 | 0.0005 | 0.0608 | 0.0177 | 0 | 0.0001 | 0.0118 | 0.37 | 0 | 0.0377 | 0.6279 | 0.7069 |
| 10523 | CHERP | -1.73 | -1.11 | 0.21 | -0.93 | 0.62 | -0.67 | -1.09 | -2.15 | -3.27 | -1.95 | -1.59 | -2.37 | 0 | 0.0426 | 0.7724 | 0.0033 | 0.3027 | 0.0163 | 0 | 0 | 0.0001 | 0.2035 | 0 | 0 |
| 1187 | CLCNKA | 0.55 | 0.78 | 3.4 | 2.79 | 3.59 | 3.16 | 3.75 | 3.4 | 2.17 | 1.1 | 1.52 | 1.65 | 0.03 | 0.0902 | 0 | 0.0092 | 0 | 0 | 0 | 0 | 0 | 0 | 0 | 0.105 |
| 10664 | CTCF | -0.72 | -0.16 | -0.47 | -0.48 | -0.88 | -2 | -2.51 | -3.27 | 0.62 | -1.51 | -1.78 | -1.96 | 0.1386 | 0.6369 | 0.6375 | 0.6168 | 0.0395 | 0 | 0.0416 | 0 | 0.4482 | 0.072 | 0.0885 | 0.1062 |
| 2017 | CTTN | -1.25 | -1.12 | -0.05 | -1.44 | -1.8 | -2.6 | -2.59 | -1.77 | -1.96 | -1.88 | -0.37 | -1.08 | 0.2031 | 0.0931 | 0.9534 | 0.3748 | 0.3402 | 0.016 | 0.0264 | 0.0071 | 0.0139 | 0.1165 | 0.6178 | 0.003 |
| 1663 | DDX11 | -1.23 | -2 | -1.84 | -1.42 | -2.46 | -1.68 | -2.68 | -2.66 | -4.18 | 0.1 | -0.95 | -1.13 | 0.2502 | 0.0347 | 0.0551 | 0.3653 | 0.0975 | 0.0994 | 0.0093 | 0.0154 | 0.05 | 0.9169 | 0.5568 | 0.2938 |
| 9416 | DDX23 | -1.95 | -0.84 | -1.7 | -1.28 | -2.68 | -3.29 | -4.68 | -2.54 | -0.09 | 0.22 | -1.91 | -1.54 | 0 | 0.1374 | 0.2556 | 0.0683 | 0.0355 | 0 | 0 | 0.0002 | 0.9316 | 0.6204 | 0.0584 | 0.3388 |
| 162989 | DEDD2 | -1.09 | -0.69 | 2.67 | 0.65 | 1.33 | 3.24 | 3.96 | 4.42 | 0.3 | 3.33 | 3.91 | 1.15 | 0.069 | 0.4763 | 0.2743 | 0.8447 | 0.5246 | 0.0007 | 0 | 0 | 0.7818 | 0.0094 | 0 | 0.3832 |
| 8562 | DENR | -2.17 | -3.73 | -2.03 | -2.55 | -0.53 | -3.83 | -2.36 | -0.66 | 0.51 | 3.01 | -0.66 | -0.54 | 0.001 | 0.0037 | 0.0649 | 0.0117 | 0.7599 | 0.0095 | 0.1161 | 0.4388 | 0.7097 | 0.0729 | 0.8266 | 0.5682 |
| 1676 | DFFA | -2.26 | -1.45 | -0.17 | -4.05 | -0.6 | -1.82 | -2.98 | -2.77 | -4.38 | -1.57 | 2.02 | -0.34 | 0.0294 | 0.0317 | 0.9358 | 0.0029 | 0.0259 | 0.2044 | 0.049 | 0.0013 | 0 | 0.1071 | 0.2031 | 0.8533 |
| 57647 | DHX37 | -0.12 | 0.06 | -0.84 | 0.5 | 0.98 | -0.53 | -0.04 | -0.83 | -1.39 | -1.41 | -0.43 | 0.38 | 0.6338 | 0.7799 | 0.0625 | 0.0438 | 0.008 | 0.2323 | 0.9202 | 0.141 | 0.3738 | 0 | 0.7502 | 0.6686 |
| 23405 | DICER1 | 0.11 | -0.18 | -0.78 | -0.32 | -0.95 | -1.36 | -0.63 | -1.05 | -0.15 | -0.1 | -0.62 | -2.09 | 0.8077 | 0.5938 | 0.1395 | 0.376 | 0.0256 | 0.0238 | 0.1859 | 0.0403 | 0.8398 | 0.4948 | 0.3699 | 0.0306 |
| 1755 | DMBT1 | -0.35 | -1.35 | -2.01 | -2.4 | -1.18 | -2.4 | -2.04 | -2.35 | -2.62 | -1.13 | -0.97 | -1.63 | 0.2077 | 0.0024 | 0.0695 | 0 | 0 | 0 | 0 | 0 | 0 | 0 | 0.0633 | 0.0096 |
| 1847 | DUSP5 | 0.63 | 2.1 | 4.22 | 2.8 | 2.48 | 3.66 | 3.06 | 3.99 | 3.12 | 3.17 | 1.9 | 2.35 | 0.0293 | 0.0581 | 0.001 | 0.0174 | 0.0133 | 0.0002 | 0 | 0 | 0.0001 | 0 | 0 | 0 |
| 1783 | DYNC1LI2 | -1.56 | -0.92 | -0.42 | -4.59 | -0.99 | -2.11 | -3.35 | -3.89 | 1.25 | -3.72 | -3.57 | -1.85 | 0.0419 | 0.2337 | 0.6861 | 0.0265 | 0.3005 | 0.0005 | 0.0014 | 0.0011 | 0.4797 | 0.0994 | 0.0365 | 0.6133 |
| 1874 | E2F4 | -2.15 | -1.59 | -0.98 | -0.54 | -2.6 | -1.06 | -1.4 | -1.91 | -3.34 | 1.95 | 0.79 | -1.4 | 0 | 0.0865 | 0.4252 | 0.374 | 0.0022 | 0.2206 | 0.016 | 0 | 0.0007 | 0.3581 | 0.4726 | 0.1651 |
| 10289 | EIF1B | -2.44 | -3.62 | -0.09 | 0.75 | -0.83 | -1.13 | -3.01 | -2.33 | -2.44 | 2.05 | 0.86 | -1.54 | 0.0028 | 0.0589 | 0.9223 | 0.6115 | 0.7179 | 0.3878 | 0.2729 | 0.0002 | 0.0007 | 0.1577 | 0.7924 | 0.4939 |
| 26523 | EIF2C1 | -1.1 | -1.34 | 0.33 | -0.23 | -0.77 | -1.91 | -2.45 | -2.26 | -1.75 | -0.61 | -0.77 | -1.94 | 0 | 0 | 0.403 | 0.5491 | 0.0027 | 0.0073 | 0 | 0.0006 | 0 | 0.1088 | 0 | 0 |
| 192669 | EIF2C3 | 0.15 | -1.56 | 0.21 | -0.67 | -0.75 | -0.01 | -0.55 | -0.17 | -0.32 | 0.15 | 0.01 | 0.04 | 0.6966 | 0.0253 | 0.4382 | 0.0368 | 0.4028 | 0.9907 | 0.0031 | 0.5691 | 0.2291 | 0.6929 | 0.9899 | 0.9143 |
| 1977 | EIF4E | 0.5 | 0.58 | 0 | -0.21 | 0.45 | 0.2 | 0.43 | 0.06 | -0.07 | -0.42 | -0.04 | 1.04 | 0.3286 | 0.0244 | 0.9837 | 0.6536 | 0.3854 | 0.6697 | 0.2304 | 0.5772 | 0.9077 | 0.0078 | 0.8801 | 0.0008 |
| 56478 | EIF4ENIF1 | -1.02 | -0.87 | -0.61 | -0.38 | -1.15 | 0.1 | -1.32 | -2.3 | -2.69 | 0.28 | -0.98 | -1.93 | 0.0003 | 0.1116 | 0.5059 | 0.7353 | 0.2657 | 0.8876 | 0.3404 | 0 | 0.0053 | 0.7672 | 0.575 | 0.2191 |
| 8672 | EIF4G3 | -1.12 | 0.42 | 0.5 | -0.49 | 1.39 | -0.92 | -2.25 | -2.2 | 0.26 | -1.06 | -1.08 | -1.13 | 0.2811 | 0.4605 | 0.7288 | 0.6427 | 0.0309 | 0.2864 | 0.0077 | 0.0246 | 0.8953 | 0.0897 | 0.4269 | 0.5823 |
| 1994 | ELAVL1 | -1.54 | -1.4 | -0.9 | -1.41 | -0.61 | -0.46 | -1.13 | -2.69 | -2.85 | -0.99 | -0.45 | -2.11 | 0 | 0.0003 | 0.1454 | 0 | 0.5127 | 0.5017 | 0.0185 | 0 | 0 | 0.1385 | 0.1767 | 0.0121 |
| 2067 | ERCC1 | -2.24 | -4.76 | -4.81 | -3.87 | -6.78 | -3.16 | -2.45 | -2.04 | -3.87 | -0.74 | 4.41 | 0.01 | 0 | 0.0086 | 0.0007 | 0.0004 | 0 | 0.0003 | 0.0012 | 0.0103 | 0.0056 | 0.7926 | 0.0555 | 0.9985 |
| 2067 | ERCC1 | 0.34 | 1.43 | 1.83 | 2.54 | 3.01 | 4.76 | 2.65 | 1.88 | 0.48 | 1.94 | 1 | 1.34 | 0.3125 | 0.141 | 0.0921 | 0.0166 | 0 | 0 | 0 | 0.0054 | 0.1511 | 0.3408 | 0.321 | 0.0876 |
| 10961 | ERP29 | 1.97 | 0.3 | -0.72 | -1.03 | -3.32 | -2.44 | -1.4 | 0.63 | -0.55 | -2.33 | -1.8 | 2.82 | 0.0036 | 0.6781 | 0.5992 | 0.0001 | 0 | 0.0106 | 0.1205 | 0.026 | 0.7552 | 0.0309 | 0.4531 | 0.5139 |
| 57488 | FAM62B | -0.1 | 0.06 | 1.35 | -1.43 | 1.08 | -1.79 | -3.83 | -2.49 | 0.4 | 0.42 | 0.06 | -0.75 | 0.8794 | 0.929 | 0.0033 | 0.0005 | 0.1849 | 0.2742 | 0.0034 | 0 | 0.4028 | 0.5816 | 0.9654 | 0.7704 |
| 81610 | FAM83D | -2.1 | -1.88 | -0.41 | -0.62 | -1.72 | -2.5 | -2.73 | -4.62 | -1.91 | -1.27 | -2.08 | -2.95 | 0 | 0.04 | 0.6727 | 0.2305 | 0.2029 | 0.0332 | 0.1209 | 0 | 0.0011 | 0.1195 | 0.2996 | 0.2596 |
| 25793 | FBXO7 | 0.04 | 0.49 | 1.04 | 3.05 | 5.54 | 3.38 | 2.94 | 1.55 | 1.19 | 2.46 | 1.22 | 1.06 | 0.9169 | 0.3006 | 0.0807 | 0.0001 | 0 | 0 | 0 | 0.0337 | 0.0308 | 0.2084 | 0.0189 | 0.5215 |
| 26190 | FBXW2 | -1.49 | 0.02 | -1.49 | -2.92 | -2.98 | -3.11 | -2.38 | -1.87 | -1.05 | 0.45 | -0.81 | -2.53 | 0.001 | 0.9524 | 0.2001 | 0 | 0.0005 | 0 | 0 | 0 | 0.0794 | 0.6558 | 0.2028 | 0 |
| 2295 | FOXF2 | -2.4 | -1.34 | -2.07 | -1.76 | -3.28 | -2.01 | -3.85 | -3.41 | -2.4 | 0.55 | -1.26 | -2.32 | 0.0002 | 0.0035 | 0.0646 | 0.1222 | 0.0183 | 0.0137 | 0.0347 | 0 | 0.0038 | 0.5001 | 0.0371 | 0.0727 |
| 55810 | FOXJ2 | -3.14 | -1.74 | -0.17 | -2.8 | 0.24 | -3.14 | -3.01 | -1.53 | -0.9 | -0.76 | -1.22 | -0.6 | 0 | 0.0004 | 0.9303 | 0 | 0.8767 | 0.0005 | 0.0142 | 0.0042 | 0.0843 | 0.452 | 0.0647 | 0.5367 |
| 2309 | FOXO3 | -1.4 | -0.23 | -0.88 | -0.93 | -0.47 | -2.03 | -2.64 | -3.76 | -0.52 | -2.16 | -2.24 | -1.86 | 0.0335 | 0.3884 | 0.0978 | 0.3294 | 0.4077 | 0.0297 | 0.0863 | 0 | 0.5787 | 0.0027 | 0.1758 | 0.2912 |
| 2309 | FOXO3 | -3.44 | -1.08 | 0.19 | -3.71 | -0.11 | -3.27 | -3.34 | -2.79 | 1.39 | -2.44 | -2.46 | -1.65 | 0.0087 | 0.1848 | 0.9129 | 0.0008 | 0.9233 | 0.0105 | 0.1973 | 0.0329 | 0.3353 | 0.2633 | 0.0471 | 0.2701 |
| 80020 | FOXRED2 | -3.69 | -2.66 | -1.42 | -5.14 | -3.39 | -3.85 | -4.05 | -3.91 | -3.56 | -2 | 0.2 | -2.2 | 0 | 0 | 0.4162 | 0.005 | 0.0742 | 0 | 0 | 0 | 0 | 0.0489 | 0.8945 | 0 |
| 80020 | FOXRED2 | -0.25 | -0.08 | -1.77 | -1.68 | -1.63 | -2.02 | -2.68 | -3.17 | -3.79 | -0.66 | 0.82 | -0.87 | 0.2389 | 0.931 | 0 | 0.2278 | 0.0552 | 0.2196 | 0.0382 | 0 | 0.0013 | 0.5772 | 0.5578 | 0.5789 |
| 2622 | GAS8 | -1.66 | -2 | -0.67 | -0.81 | -2.15 | -2.31 | -3.38 | -3.36 | -2.38 | 1.41 | -0.59 | -1.14 | 0.0014 | 0.0038 | 0.3164 | 0.702 | 0.3437 | 0.1865 | 0.0073 | 0.0004 | 0.0475 | 0.2178 | 0.694 | 0.4238 |
| 9518 | GDF15 | 3.65 | 3.73 | 3.44 | 3.19 | 2.52 | 2.98 | 2.98 | 2.65 | 2.72 | -0.59 | 0.99 | 3.36 | 0 | 0 | 0 | 0.0033 | 0 | 0.0123 | 0.0215 | 0.0183 | 0.2194 | 0.7019 | 0.7295 | 0 |
| 54552 | GNL3L | -3.19 | -3.12 | -1.09 | -5.44 | -0.57 | -2.72 | -3.79 | -4.42 | -5.77 | -2.07 | 1.79 | -1.86 | 0.2143 | 0.0636 | 0.73 | 0.0003 | 0.4311 | 0.0657 | 0.0221 | 0.08 | 0.0001 | 0.0058 | 0.1756 | 0.3772 |
| 2805 | GOT1 | -3.13 | -4.48 | -2.59 | -2.8 | -4.34 | -4.12 | -5.83 | -4.92 | -2.58 | -4.34 | -4.56 | -1.85 | 0 | 0 | 0 | 0.0162 | 0.0007 | 0.0019 | 0.0201 | 0 | 0 | 0.0017 | 0.0002 | 0 |
| 222487 | GPR97 | 0.8 | 1.06 | 7.71 | 7.47 | 2.9 | 1.03 | 1.01 | 0.81 | 0.28 | 1.48 | 1.79 | 1.38 | 0 | 0.0038 | 0 | 0 | 0 | 0.2749 | 0.1436 | 0.1522 | 0.641 | 0.3052 | 0 | 0.171 |
| 2962 | GTF2F1 | -1.71 | -1.5 | 0.26 | -1.85 | -0.5 | -1.32 | -1.99 | -3.49 | -2.68 | -2.68 | 0.41 | -1.86 | 0 | 0.0728 | 0.77 | 0.0002 | 0.7511 | 0.1422 | 0.0163 | 0 | 0.0013 | 0.0849 | 0.7932 | 0.2082 |
| 2971 | GTF3A | -0.62 | -1.47 | 0.18 | 1.11 | 0 | -2.8 | -3.47 | -2.67 | -1.71 | 3.08 | -0.41 | 0.74 | 0.6608 | 0.1897 | 0.957 | 0.5973 | 0.9996 | 0.024 | 0.0333 | 0.0807 | 0.0101 | 0.31 | 0.911 | 0.765 |
| 9555 | H2AFY | -3.59 | -2.58 | -1.33 | -1.94 | -2.29 | -2.5 | -2.97 | -3.48 | -2.27 | -0.24 | -2.03 | -2.59 | 0 | 0.0009 | 0.2382 | 0.0779 | 0.2252 | 0.0395 | 0.041 | 0 | 0.0236 | 0.5503 | 0.0034 | 0.2502 |
| 54919 | HEATR2 | 0.64 | 1.34 | -0.7 | 0.19 | 0.3 | 0.13 | -0.72 | -0.13 | 0.87 | -1.07 | 0.34 | 0.47 | 0.0003 | 0 | 0.1628 | 0.7625 | 0.1808 | 0.7977 | 0.0758 | 0.5329 | 0.0217 | 0.0002 | 0.2185 | 0.2376 |
| 124790 | HEXIM2 | -0.74 | -0.46 | 3.56 | 1.93 | 1.83 | 3.53 | 3.25 | 3.3 | -0.54 | 2.88 | 3.26 | 1.6 | 0 | 0.4923 | 0.0209 | 0.394 | 0.2792 | 0 | 0.0004 | 0 | 0.6027 | 0.0088 | 0 | 0.0882 |
| 3006 | HIST1H1C | 0.11 | 1.03 | 3.51 | -0.27 | 3.63 | 3.12 | 4.88 | 5.23 | 1.11 | 3.72 | 3.31 | 1.37 | 0.8834 | 0.1381 | 0.2294 | 0.9422 | 0.0318 | 0.0387 | 0.0343 | 0 | 0.2757 | 0.0002 | 0.2236 | 0 |
| 9987 | HNRPDL | -2.35 | -0.42 | -0.42 | -1.02 | 0.05 | -3.18 | -3.33 | -2.15 | -1.01 | 0 | -0.01 | -1.84 | 0 | 0.4096 | 0.6601 | 0.4892 | 0.8748 | 0.0058 | 0.0124 | 0.0016 | 0.4511 | 0.999 | 0.9948 | 0 |
| 3217 | HOXB7 | -2.41 | -0.76 | -0.19 | -2.68 | -3.53 | -2.9 | -4.82 | -3.71 | -3.56 | 1.34 | 2.14 | -1 | 0.0312 | 0.4345 | 0.8705 | 0.3002 | 0.0308 | 0.01 | 0.0022 | 0.0015 | 0 | 0.3879 | 0.144 | 0.1304 |
| 3223 | HOXC6 | -2.74 | -2.72 | -1.2 | -2.97 | -2.32 | -4.3 | -3.3 | -1.87 | -2.57 | 1.64 | 1.26 | -0.63 | 0.0002 | 0.0204 | 0.3091 | 0.2922 | 0.0204 | 0 | 0.0017 | 0.0006 | 0.0008 | 0.3485 | 0.4884 | 0.7392 |
| 3397 | ID1 | 0.74 | 2.3 | 3.51 | -0.24 | 2.01 | 4.01 | 4.08 | 4.57 | 1.7 | 0.11 | 1.25 | 1.32 | 0.3967 | 0.0138 | 0.0342 | 0.9129 | 0.0653 | 0.0002 | 0.2369 | 0 | 0.1642 | 0.9235 | 0.4647 | 0.0717 |
| 3398 | ID2 | 0.57 | 2.96 | 7.17 | 2.53 | 4.77 | 2.47 | 3 | 3.46 | 3.45 | 3.08 | 2.54 | 1.35 | 0.6024 | 0.0148 | 0 | 0.1899 | 0.0155 | 0.0458 | 0.2423 | 0 | 0 | 0.0163 | 0.0101 | 0.6353 |
| 3619 | INCENP | 0.41 | 0.24 | -0.67 | -0.68 | 0.33 | -0.48 | 0.12 | -2.73 | -4.26 | -1.45 | -0.17 | -1.95 | 0.2763 | 0.5965 | 0.332 | 0.1995 | 0.3648 | 0.2283 | 0.8737 | 0 | 0.0056 | 0.239 | 0.8251 | 0.0317 |
| 27130 | INVS | -1.42 | -1.29 | 0.82 | -2.43 | -2.54 | -4.4 | -3.45 | -2.87 | -0.66 | 0.88 | 0 | -1.99 | 0.0122 | 0.1483 | 0.1919 | 0.1075 | 0.2322 | 0 | 0.0239 | 0 | 0.4952 | 0.5179 | 0.9996 | 0.0321 |
| 81618 | ITM2C | 0.93 | 1.45 | 3.1 | 1.67 | 2.05 | 3.61 | 3.35 | 3.8 | 1.87 | 2.53 | 2.33 | 1.59 | 0.0003 | 0.048 | 0 | 0.04 | 0 | 0.0111 | 0 | 0 | 0.0243 | 0 | 0.0008 | 0 |
| 81621 | KAZALD1 | -1.96 | -2.35 | -1.03 | -0.79 | -3.31 | -1.52 | -3.33 | -3.66 | -3.93 | 0.85 | -0.99 | -1.23 | 0.0001 | 0.0247 | 0.3115 | 0.5219 | 0.0449 | 0.3609 | 0.0003 | 0.0462 | 0.0574 | 0.5668 | 0.3337 | 0.5166 |
| 10945 | KDELR1 | 2.16 | 0.97 | 0.86 | 3.11 | 2.55 | 4.1 | 1.9 | 1.37 | -1.15 | 2.3 | 1.88 | 2.42 | 0.0835 | 0.428 | 0.5129 | 0.0002 | 0 | 0 | 0.0255 | 0 | 0.1566 | 0.2989 | 0.2983 | 0.0262 |
| 9817 | KEAP1 | -1.81 | 0.26 | -2.77 | -2.71 | -2.93 | -1.47 | -0.54 | -0.73 | -1.37 | -0.01 | 0.09 | -1.42 | 0 | 0.5383 | 0.0221 | 0.0265 | 0.0035 | 0.0116 | 0.2926 | 0.0117 | 0.3763 | 0.9748 | 0.9322 | 0.3744 |
| 10657 | KHDRBS1 | -1.33 | -1.86 | 0.64 | 0.69 | -0.55 | -2.81 | -4.31 | -2.54 | 0.67 | -2.59 | -3.14 | -0.91 | 0.0223 | 0.2225 | 0.668 | 0.3157 | 0.7957 | 0.1036 | 0.0358 | 0.0028 | 0.1259 | 0.1645 | 0.0175 | 0.4629 |
| 57292 | KIR2DL5A | 0.31 | -0.18 | 0.64 | -0.13 | 0.63 | 0.29 | 0.02 | 1.22 | 0.56 | -0.88 | -0.06 | 0.36 | 0.511 | 0.6026 | 0.1505 | 0.758 | 0.173 | 0.1282 | 0.9793 | 0.0001 | 0 | 0.0001 | 0.8517 | 0.0125 |
| 4254 | KITLG | 0.6 | 1.25 | 2.43 | 5.07 | 4.27 | 0.42 | 2.37 | 2.94 | 5.64 | 4.24 | 1.26 | 0.3 | 0.501 | 0.0004 | 0.0229 | 0.0889 | 0.1024 | 0.9059 | 0.2486 | 0.0008 | 0.0004 | 0.0596 | 0.7846 | 0.9383 |
| 10365 | KLF2 | -3.59 | -4.65 | -3.92 | -3.71 | -3.52 | -1.95 | -2.25 | -2.75 | -4.67 | -0.67 | 0.39 | -2.43 | 0.0002 | 0.0001 | 0.1145 | 0.1648 | 0.0446 | 0.0171 | 0.286 | 0.0074 | 0 | 0.4936 | 0.2066 | 0 |
| 1316 | KLF6 | -2.6 | -0.09 | -2.39 | -4.35 | -2.13 | -4.06 | -3.39 | -3.51 | -1.73 | -1.86 | -1.09 | -2.06 | 0.0014 | 0.8178 | 0.1161 | 0.0023 | 0.0057 | 0 | 0.0429 | 0 | 0.1776 | 0.0019 | 0.6279 | 0.5217 |
| 1316 | KLF6 | -2.72 | -0.7 | -1.15 | -4.23 | -3.03 | -4.35 | -3.13 | -3.98 | -2.49 | -0.62 | -0.96 | -1.92 | 0 | 0.5671 | 0.1508 | 0.0092 | 0 | 0.0028 | 0.0005 | 0 | 0.0128 | 0.5732 | 0.6182 | 0.2298 |
| 283212 | KLHL35 | -3.7 | -3.39 | -3.89 | -4.74 | -4.96 | -2.06 | -2.2 | -1.81 | -3.95 | -3.27 | -1.34 | -1.06 | 0 | 0 | 0.0562 | 0.003 | 0 | 0.0109 | 0.0236 | 0 | 0 | 0.0356 | 0.5737 | 0.0003 |
| 25875 | LETMD1 | -2.7 | -3.06 | -1.94 | 1.34 | -0.93 | -1.1 | -1.44 | -2.82 | -1.9 | -0.43 | -1.84 | -2.17 | 0.005 | 0 | 0.107 | 0.4556 | 0.273 | 0.1093 | 0.1111 | 0 | 0.0156 | 0.6923 | 0.1613 | 0.3949 |
| 4111 | MAGEA12 | 0.4 | 0.66 | 1.14 | 2.07 | 1.88 | 0.95 | 1.95 | 2.03 | 4 | 0.28 | 0.72 | 1.18 | 0.6992 | 0.289 | 0.2306 | 0 | 0 | 0.0884 | 0.0114 | 0.0167 | 0 | 0.746 | 0.4989 | 0.6218 |
| 28985 | MCTS1 | -1.16 | 0.96 | 5.39 | 2.06 | 3.39 | 2.28 | 3.25 | 3.08 | 1.14 | 3.88 | 2.89 | 1.53 | 0.3139 | 0.4239 | 0.0298 | 0.4877 | 0.1355 | 0.1356 | 0.0813 | 0.0072 | 0.4844 | 0.0191 | 0.1578 | 0.3847 |
| 112950 | MED8 | -2.1 | -1.4 | -0.26 | -1.22 | -3.07 | -1.94 | -2.92 | -3.66 | -1.65 | -1.66 | -2.21 | -2.88 | 0 | 0 | 0.6333 | 0.0164 | 0.0004 | 0.0434 | 0.0001 | 0 | 0 | 0.0019 | 0.1669 | 0.2095 |
| 9258 | MFHAS1 | -1.47 | -0.04 | -0.37 | -4.18 | -2.38 | -2.6 | -2.82 | -3.4 | -2.1 | 0.41 | -1.27 | -2.18 | 0.0153 | 0.9218 | 0.6905 | 0.0015 | 0.0233 | 0.0008 | 0.0973 | 0 | 0.033 | 0.4966 | 0.2587 | 0 |
| 4256 | MGP | 1.18 | -0.11 | 4.57 | 3.66 | 4.99 | 4.2 | 1.46 | 3.31 | 0.37 | 3.31 | 4.98 | 2.44 | 0.0983 | 0.9495 | 0.0064 | 0.0008 | 0.0056 | 0.0001 | 0.5731 | 0 | 0.6163 | 0 | 0 | 0.0774 |
| 574469 | MIR519B | 0.44 | 0.8 | -0.61 | 0.96 | 0.78 | 0.25 | 0.43 | 0.67 | 0.62 | -0.73 | -0.17 | 0.29 | 0.3374 | 0.0059 | 0.3437 | 0.1233 | 0.4083 | 0.2766 | 0.402 | 0.0001 | 0.0327 | 0.0178 | 0.4023 | 0.5689 |
| 407052 | MIR95 | 0.7 | 0.28 | 0.53 | 0.29 | 0.08 | -0.12 | -0.15 | 0.5 | 0.66 | -0.73 | -0.35 | 0.22 | 0.001 | 0.3792 | 0.0424 | 0.4977 | 0.8493 | 0.6399 | 0.4321 | 0.0207 | 0 | 0.0487 | 0.3283 | 0.7395 |
| 23609 | MKRN2 | -2.6 | -2.58 | -1.66 | -1.91 | -2.03 | -3.44 | -4.09 | -2.45 | -0.4 | -0.61 | -2.78 | -2.58 | 0.0003 | 0 | 0.0327 | 0.081 | 0.1854 | 0 | 0.0164 | 0 | 0.3427 | 0.5155 | 0.0026 | 0.1644 |
| 9757 | MLL4 | -0.89 | -0.89 | -0.69 | -1.6 | -0.87 | -0.76 | -1.48 | -1.93 | -3.51 | -1.39 | -0.32 | -1.31 | 0.0058 | 0.1379 | 0.4154 | 0.1193 | 0.0099 | 0.067 | 0.1659 | 0.0054 | 0.0341 | 0.0402 | 0.8696 | 0.0055 |
| 124540 | MSI2 | -0.36 | 1.25 | 3.56 | 2.09 | 2.58 | 2.86 | 2.16 | 1.97 | -0.61 | 1.65 | 2.51 | 1.66 | 0.6725 | 0.0257 | 0 | 0.0293 | 0.0001 | 0 | 0.0397 | 0.0247 | 0.2433 | 0.0092 | 0 | 0 |
| 10232 | MSLN | 3.93 | 3.35 | 1.21 | 2.16 | 1.7 | 4.11 | 3.44 | 3.1 | 1.65 | -2.47 | 1.67 | 2.71 | 0 | 0.0041 | 0.3005 | 0.1345 | 0.1386 | 0 | 0 | 0.0003 | 0.0243 | 0.115 | 0.5395 | 0.386 |
| 9112 | MTA1 | -0.81 | -1.04 | -2.93 | -0.24 | -2.09 | -0.98 | -1.86 | -2.55 | -4.31 | 0.24 | 1.15 | 0.37 | 0.5243 | 0.173 | 0.2157 | 0.9142 | 0.3788 | 0.5692 | 0.2993 | 0 | 0.0353 | 0.8165 | 0.6173 | 0.5086 |
| 84939 | MUM1 | -2.11 | -1.76 | 0.23 | -0.37 | -1.77 | -0.42 | -2.03 | -2.25 | -3.29 | -0.69 | 0.9 | -1.52 | 0 | 0.0259 | 0.8689 | 0.8305 | 0.2902 | 0.6186 | 0.002 | 0.014 | 0.0058 | 0.1076 | 0.0652 | 0 |
| 83463 | MXD3 | -1.9 | -1.56 | -2.52 | -3.87 | -5.25 | -0.74 | -3.3 | -3.03 | -4.14 | -0.94 | 0.25 | -0.28 | 0 | 0.3359 | 0.0121 | 0.0005 | 0.0008 | 0.6673 | 0 | 0.231 | 0.0055 | 0.002 | 0.4222 | 0.9096 |
| 4676 | NAP1L4 | -0.67 | -0.3 | -1.51 | -1 | -3.44 | -1.72 | -2.26 | -2.1 | 0.77 | -2.27 | -1.12 | -0.8 | 0.2811 | 0.6874 | 0.0006 | 0.1564 | 0 | 0 | 0.0027 | 0.0729 | 0.3699 | 0.008 | 0.4099 | 0.0186 |
| 55226 | NAT10 | -1.42 | -0.05 | 0.8 | -1.37 | 0.39 | -1.28 | -2.05 | -3.3 | -1.81 | -3.65 | -1.85 | -2.3 | 0 | 0.9295 | 0.0915 | 0.1358 | 0.1262 | 0.0026 | 0.0455 | 0 | 0 | 0.0972 | 0.2837 | 0.3411 |
| 4790 | NFKB1 | -2.92 | -2.14 | 0.68 | -3.22 | -2.06 | -4.63 | -4.1 | -2.99 | 0.9 | -1.43 | -2.49 | -2.28 | 0 | 0.0454 | 0.128 | 0 | 0.3261 | 0 | 0.0716 | 0.0002 | 0.36 | 0.2983 | 0.0439 | 0 |
| 8602 | NOL14 | 0.6 | 1.1 | -0.06 | -0.68 | -1.93 | -0.57 | -1.04 | -2.29 | -3.13 | -1.7 | 0.25 | -0.42 | 0.6449 | 0.0256 | 0.9357 | 0.1625 | 0.0476 | 0.0323 | 0.0513 | 0 | 0 | 0.2963 | 0.8565 | 0.8929 |
| 8021 | NUP214 | -1.99 | -0.55 | 0.85 | -0.17 | -1.15 | -0.04 | -0.48 | -1.94 | -3.96 | -3.16 | -0.87 | -2.56 | 0.0001 | 0.4577 | 0.0181 | 0.8358 | 0.4581 | 0.9249 | 0.3025 | 0 | 0.002 | 0.0675 | 0.3083 | 0.1236 |
| 4957 | ODF2 | -1.07 | -2.96 | 0.44 | -0.78 | -1.72 | -2.31 | -3.6 | -2.53 | -3.35 | -0.34 | -1.51 | -2.07 | 0.0872 | 0.0078 | 0.6186 | 0.7128 | 0.2286 | 0.0976 | 0.0124 | 0.002 | 0.0018 | 0.8164 | 0.3831 | 0 |
| 11339 | OIP5 | -0.75 | -2.28 | 1.22 | 0.6 | -1.18 | -1.06 | -3.04 | -3.54 | -2.9 | -0.13 | -1.56 | -1.1 | 0.0644 | 0.009 | 0.2664 | 0.6398 | 0.5001 | 0.3623 | 0.1476 | 0 | 0.0002 | 0.8451 | 0.5345 | 0.6879 |
| 11339 | OIP5 | -3.68 | -5.92 | -0.31 | -1.72 | -1.84 | -2.05 | -3.56 | -2.98 | -2.91 | -0.49 | -1.63 | -1.73 | 0 | 0 | 0.8108 | 0.272 | 0.0873 | 0.0242 | 0.0815 | 0 | 0 | 0.3896 | 0.2552 | 0.3764 |
| 55074 | OXR1 | -2.86 | -5.82 | -1.36 | -4.31 | -1.6 | -3.64 | -3.5 | -2.71 | 1.45 | 0.48 | -2.33 | -2.16 | 0 | 0 | 0.1504 | 0 | 0.3599 | 0.001 | 0.0081 | 0.0003 | 0.3264 | 0.4465 | 0.1381 | 0 |
| 5032 | P2RY11 | -1.89 | -1.22 | -0.28 | -1.03 | -0.39 | 0.72 | -0.4 | -1.19 | -3.01 | -1.9 | 0.69 | -0.03 | 0.0179 | 0 | 0.9008 | 0.4661 | 0.5982 | 0.3197 | 0.7462 | 0 | 0 | 0.0168 | 0.6629 | 0.9885 |
| 5036 | PA2G4 | -0.71 | -3.56 | -2.36 | -4.06 | -4.22 | -3.3 | -5.61 | -5.56 | -3.35 | -2.56 | -0.2 | 1.51 | 0.5467 | 0.0664 | 0.1198 | 0.0164 | 0.0069 | 0.1441 | 0.0087 | 0 | 0.0299 | 0.2112 | 0.9454 | 0.0943 |
| 54623 | PAF1 | -2.47 | -0.47 | -0.1 | -2.02 | -1.21 | -2.79 | -3.94 | -2.73 | -2.97 | -1.31 | -0.44 | -0.15 | 0.0304 | 0.2837 | 0.649 | 0.0593 | 0.4324 | 0.0267 | 0.0002 | 0.0093 | 0.0002 | 0.0828 | 0.1169 | 0.9076 |
| 5058 | PAK1 | -2.07 | -0.89 | -2.34 | -2.92 | -3.12 | -2.87 | -3.49 | -0.73 | -1.82 | 1.34 | 0.64 | -1.36 | 0 | 0.0056 | 0.0001 | 0.0273 | 0.0772 | 0.0001 | 0.044 | 0.3569 | 0.0098 | 0.3973 | 0.6764 | 0.4133 |
| 5064 | PALM | 0.22 | 2.87 | 1.05 | 1.15 | 2.33 | 2.75 | 4.27 | 3.35 | 1.27 | 2.11 | 5.24 | 3.23 | 0.7919 | 0.0003 | 0.729 | 0.4322 | 0.0031 | 0.0077 | 0.04 | 0 | 0.079 | 0.0221 | 0 | 0.0291 |
| 23598 | PATZ1 | 0.33 | -0.11 | -1.93 | -0.85 | -0.28 | -0.89 | -0.63 | -2.46 | -2.9 | -1.48 | -0.83 | -0.94 | 0.4223 | 0.6433 | 0 | 0.2077 | 0.5863 | 0.2732 | 0.3205 | 0 | 0.0026 | 0 | 0.6753 | 0.2526 |
| 5092 | PCBD1 | -0.69 | 0.24 | 1.5 | -0.79 | -0.5 | 1.05 | -1.17 | -1.93 | -2.92 | 1.37 | 1.71 | -0.05 | 0.4811 | 0.8913 | 0.0658 | 0.6671 | 0.5838 | 0.0306 | 0.0003 | 0 | 0.0134 | 0.398 | 0.0519 | 0.9735 |
| 5092 | PCBD1 | -1.55 | 0.16 | 0.71 | -1.98 | -1.19 | 0.77 | -1.41 | -1.32 | -3.08 | 2.19 | 1.52 | 0.16 | 0.0722 | 0.8854 | 0.3454 | 0.3168 | 0.0166 | 0.0851 | 0.0014 | 0.0348 | 0.0003 | 0.2344 | 0.3093 | 0.8074 |
| 5094 | PCBP2 | 0.59 | 0.35 | 0.09 | 0.64 | 0.63 | 0.24 | 0.6 | 0.64 | 0.64 | -0.75 | -0.33 | 0.49 | 0.0553 | 0.3815 | 0.7669 | 0.2662 | 0.284 | 0.48 | 0.0687 | 0.0402 | 0.0039 | 0.0593 | 0.2254 | 0.0897 |
| 9141 | PDCD5 | -2.34 | -3.51 | -1.73 | -1.69 | -2.98 | -2.87 | -5.03 | -3.78 | -3.63 | 1.91 | 2.24 | -2.08 | 0.0172 | 0.0803 | 0.3032 | 0.3796 | 0.0005 | 0 | 0.0245 | 0.0008 | 0 | 0.3274 | 0.4314 | 0.0641 |
| 5136 | PDE1A | 0.26 | 1.78 | 2.51 | 1.94 | 3.21 | 1.52 | 2.92 | 3.85 | 4.67 | 3.23 | 1.08 | 1.33 | 0.7196 | 0.1157 | 0.0033 | 0.0273 | 0 | 0.0546 | 0 | 0 | 0.0003 | 0.0056 | 0.419 | 0.3708 |
| 5136 | PDE1A | -0.52 | 1.36 | 0.43 | 3.64 | 2.75 | 1.54 | 1.66 | 2.8 | 4.29 | 3.2 | 1.42 | -0.6 | 0.3746 | 0.0229 | 0.6704 | 0 | 0 | 0 | 0.0029 | 0 | 0.0032 | 0.0248 | 0.4992 | 0.7309 |
| 10455 | PECI | -2.96 | -3.94 | -1.7 | -2.69 | -3.29 | -4.63 | -1.36 | -0.13 | 0.46 | 0.52 | -2.51 | -1.76 | 0.0005 | 0 | 0.2257 | 0.0301 | 0.0261 | 0 | 0.1444 | 0.8693 | 0.763 | 0.4265 | 0.0006 | 0 |
| 27043 | PELP1 | -0.14 | -0.49 | 0.25 | -1.74 | -1.7 | -0.47 | -2.64 | -4.03 | -4.16 | -0.96 | 0.3 | 0.75 | 0.9381 | 0.7465 | 0.5035 | 0.2774 | 0.3345 | 0.7509 | 0.0006 | 0.0053 | 0.0322 | 0.5229 | 0.6237 | 0.8172 |
| 5209 | PFKFB3 | -2.05 | -0.34 | 0.29 | -0.07 | -0.95 | -2.32 | -1.98 | -2.7 | -2.53 | 0.07 | -0.08 | -2.36 | 0 | 0.5335 | 0.1944 | 0.9146 | 0.5854 | 0 | 0.0258 | 0 | 0 | 0.9182 | 0.8893 | 0.082 |
| 26147 | PHF19 | -2.47 | -2.94 | -0.71 | -2.59 | -3.37 | -2.79 | -3.94 | -2.72 | -3.42 | 1.02 | -0.46 | -0.67 | 0.005 | 0 | 0.4989 | 0.1906 | 0.0814 | 0.0103 | 0.0153 | 0 | 0.0056 | 0.5176 | 0.5926 | 0.1322 |
| 26147 | PHF19 | -3.03 | -3.97 | -2.19 | -2.53 | -4.4 | -1.56 | -1.65 | -1.98 | -3.93 | 2.71 | 0.6 | -1.66 | 0 | 0.0022 | 0.0734 | 0.2226 | 0.0001 | 0.0466 | 0.1815 | 0.0103 | 0.0007 | 0.2254 | 0.5667 | 0.1644 |
| 9088 | PKMYT1 | -1.65 | -1.75 | -6.83 | -2.55 | -4.23 | -1.02 | -0.94 | -1 | -1.66 | -0.84 | 2.82 | -1.3 | 0.0343 | 0.1746 | 0.0003 | 0.0467 | 0.0233 | 0.4964 | 0.5264 | 0.6797 | 0.5096 | 0.5336 | 0.637 | 0.6608 |
| 84271 | POLDIP3 | 0.18 | -0.19 | -0.4 | 0.23 | 0.02 | -0.03 | 0.42 | 0.81 | 0.74 | -1.07 | 0.29 | -0.01 | 0.3139 | 0.2869 | 0.6378 | 0.5748 | 0.9497 | 0.906 | 0.0365 | 0.0001 | 0 | 0.0149 | 0.5853 | 0.986 |
| 64425 | POLR1E | -2.3 | -2.3 | -2.64 | -2.95 | -1.11 | -1.65 | -1.53 | -2.82 | -3.2 | -1.26 | -1.35 | -2.1 | 0.002 | 0 | 0.0107 | 0.0136 | 0.2692 | 0.1451 | 0.2167 | 0 | 0 | 0.0997 | 0.2775 | 0.0058 |
| 64425 | POLR1E | -3.04 | -3.21 | -3.14 | -4.49 | -1.36 | -3.32 | -1.6 | -1.92 | -1.19 | -0.5 | -1.68 | -1.87 | 0 | 0 | 0.0006 | 0 | 0.0612 | 0 | 0.1673 | 0.019 | 0.206 | 0.74 | 0.0486 | 0.0453 |
| 5430 | POLR2A | -2.66 | -3.88 | -1.23 | -0.7 | -2.2 | -1.01 | -1.58 | -3.14 | -5.86 | -4.76 | -2.02 | -1.76 | 0.002 | 0.0033 | 0.2232 | 0.554 | 0.2429 | 0.5082 | 0.4142 | 0 | 0.0228 | 0.0159 | 0.4333 | 0.5327 |
| 171568 | POLR3H | -1.43 | -2.68 | -1.14 | -0.62 | -2.02 | -1.24 | -3.51 | -2.4 | -3.83 | -1.15 | -0.65 | -2.22 | 0 | 0 | 0.5927 | 0.5515 | 0.0052 | 0.2734 | 0.0004 | 0.0032 | 0 | 0.1563 | 0.2388 | 0.0041 |
| 23645 | PPP1R15A | -3.05 | -2.66 | 0.2 | -2.49 | -2.18 | -1.05 | -1.72 | -2.02 | -3.31 | 1.18 | 1.98 | -1.07 | 0 | 0.0068 | 0.888 | 0.1505 | 0.0157 | 0.273 | 0 | 0.0475 | 0.0007 | 0.6348 | 0.0334 | 0.3528 |
| 23082 | PPRC1 | -3.13 | -1.22 | 0.75 | -1.46 | -0.61 | -1.79 | -2.21 | -3.06 | -2.5 | -1.41 | -1.69 | -1.6 | 0.0044 | 0.146 | 0.4529 | 0.0863 | 0.7945 | 0.0884 | 0.0001 | 0 | 0.0102 | 0.2471 | 0.0276 | 0.354 |
| 27339 | PRPF19 | -3.07 | -2.96 | -0.13 | -3.43 | -3.4 | -2.2 | -5.35 | -1.65 | -3.73 | -0.75 | 1.53 | 0.22 | 0.0008 | 0.0009 | 0.931 | 0.1427 | 0.0141 | 0.0257 | 0 | 0.0159 | 0.004 | 0.5348 | 0.643 | 0.5926 |
| 55771 | PRR11 | -1.91 | -1.61 | -0.76 | -2.78 | -2.21 | -3.52 | -3.92 | -4.37 | -3.04 | -1.34 | 0.51 | 0.48 | 0.004 | 0.0004 | 0.1499 | 0.0045 | 0 | 0.0003 | 0.0005 | 0 | 0.0295 | 0.1928 | 0.6017 | 0.8033 |
| 8000 | PSCA | 0.64 | 0.83 | 0.26 | 1.54 | 0.64 | 1.83 | 2.19 | 2.8 | 0.5 | 1.26 | 1.83 | 1.47 | 0.1856 | 0.3527 | 0.0439 | 0.0246 | 0.0592 | 0.0373 | 0.0141 | 0 | 0.1549 | 0.0921 | 0.0377 | 0.0079 |
| 5682 | PSMA1 | -0.11 | 0.85 | 0.64 | 2.25 | 2.56 | 2.15 | 2.44 | 3.13 | 2.83 | 2.2 | 1.75 | 0.88 | 0.8955 | 0.1769 | 0.4426 | 0.0186 | 0.0007 | 0.0281 | 0.0566 | 0.0021 | 0.0125 | 0.0022 | 0.1567 | 0.7726 |
| 5698 | PSMB9 | -1.08 | -0.62 | -0.3 | -0.28 | -0.49 | -0.27 | -2.09 | -3.41 | -4.39 | -0.86 | -1.31 | -0.49 | 0 | 0.0848 | 0.6547 | 0.5775 | 0.4348 | 0.6478 | 0.0236 | 0.0031 | 0.0013 | 0.3263 | 0.4662 | 0.7836 |
| 10197 | PSME3 | -2.38 | -3.66 | -2.06 | -1.69 | -2.26 | -2.06 | -5.02 | -4.77 | -3.96 | -0.55 | -2.16 | -1.38 | 0.0031 | 0 | 0.0117 | 0.4463 | 0.3197 | 0.3723 | 0.072 | 0 | 0.0219 | 0.5955 | 0.4307 | 0.4588 |
| 9491 | PSMF1 | -3.75 | -2.61 | -0.9 | -5.04 | -2.73 | -3.17 | -3.73 | -2.32 | -1.14 | -0.73 | -1.38 | -1.64 | 0 | 0 | 0.3936 | 0.0001 | 0.0408 | 0.0003 | 0.0636 | 0.0678 | 0.3178 | 0.5286 | 0.0967 | 0.0188 |
| 84722 | PSRC1 | -3.27 | -2.21 | -1.08 | -2.64 | -3.43 | -2.86 | -3.46 | -2.45 | -2.71 | -0.91 | -2.22 | -1.02 | 0 | 0.0037 | 0.5431 | 0.2829 | 0 | 0 | 0 | 0 | 0.0003 | 0.2491 | 0 | 0.2205 |
| 11156 | PTP4A3 | 0.29 | -0.13 | -5.03 | -3.42 | -2.02 | 1.95 | 0.08 | -0.96 | -1.76 | 2.67 | 2.57 | 0.13 | 0.3472 | 0.6343 | 0 | 0.0003 | 0.0514 | 0.1398 | 0.9306 | 0.3851 | 0.1548 | 0.2334 | 0.0257 | 0.9406 |
| 10890 | RAB10 | -1.17 | -1.55 | 1.08 | -0.04 | 0.54 | -2.23 | -1.13 | 0.09 | 2.65 | 2.98 | -1.41 | -1.05 | 0.0172 | 0.2482 | 0.5684 | 0.9717 | 0.649 | 0.0006 | 0.3125 | 0.9223 | 0.0487 | 0.0162 | 0.5598 | 0.2116 |
| 64284 | RAB17 | -0.15 | 1.44 | 4.09 | 1.94 | 2.63 | 3.95 | 4.8 | 5.66 | 1.89 | 3.28 | 3.49 | 3.27 | 0.6834 | 0.0115 | 0.1231 | 0.5915 | 0.1482 | 0.0124 | 0.0126 | 0 | 0 | 0.0016 | 0.0009 | 0.0297 |
| 51715 | RAB23 | -2.72 | -4.13 | -2.19 | -1.73 | -2.7 | -3.67 | -3.3 | -1.8 | 0.79 | 2.35 | -0.82 | -1.52 | 0.0027 | 0.006 | 0.0992 | 0.0671 | 0.3242 | 0.0069 | 0.1364 | 0 | 0.6036 | 0.254 | 0.8063 | 0.1677 |
| 25837 | RAB26 | 0.15 | 1.88 | 3.48 | 2.89 | 0.97 | 3.76 | 2.92 | 2.24 | 0.06 | 3.99 | 1.88 | 2 | 0.8421 | 0.0752 | 0.0509 | 0.1265 | 0.0607 | 0.0019 | 0.0012 | 0.0635 | 0.9733 | 0.0209 | 0.0119 | 0 |
| 8934 | RAB7L1 | -2.66 | -1.09 | -1.14 | -1.68 | -3.07 | -3.61 | -3.44 | -3.67 | -2.1 | 0.14 | -1.01 | -0.74 | 0 | 0.0543 | 0.2893 | 0.1663 | 0.0358 | 0.0165 | 0.024 | 0 | 0.0005 | 0.9189 | 0.6524 | 0.729 |
| 5936 | RBM4 | -0.1 | 0.67 | -0.53 | 0.52 | 0.01 | -0.45 | -0.16 | -0.45 | -1.25 | -2.56 | -0.35 | -1.25 | 0.878 | 0.2946 | 0.2556 | 0.4193 | 0.9891 | 0.3084 | 0.8686 | 0.0084 | 0.0509 | 0.0392 | 0.9055 | 0.373 |
| 23543 | RBM9 | -2.58 | -1.39 | -1.8 | -2.46 | -1.49 | -3.3 | -3.33 | -3.43 | -2.48 | -1.78 | -0.75 | -0.8 | 0.0505 | 0.2232 | 0 | 0.0334 | 0.527 | 0.1646 | 0.1728 | 0 | 0.0002 | 0.054 | 0.7932 | 0.7363 |
| 10171 | RCL1 | -1.65 | -1.65 | -1.18 | -0.43 | -1.8 | -1.13 | -3.03 | -3.29 | -2.29 | -1.18 | -1.42 | -2.39 | 0.0096 | 0.0115 | 0.0318 | 0.2327 | 0 | 0.2085 | 0.0024 | 0 | 0 | 0.1084 | 0.2305 | 0.239 |
| 9185 | REPS2 | -0.51 | 0.61 | 3.21 | 1.35 | 2.45 | 0.86 | 2 | 3.52 | 2.96 | 2.12 | 1.77 | 1.21 | 0.3275 | 0.2647 | 0.0017 | 0.4754 | 0.0621 | 0.0266 | 0.1055 | 0 | 0 | 0 | 0.0252 | 0 |
| 5993 | RFX5 | -2.46 | -1.53 | -2.44 | -1.51 | -2.61 | -5.01 | -4.22 | -3.23 | -1.23 | -0.76 | -2.38 | -2.13 | 0.0002 | 0.1755 | 0.1126 | 0.5602 | 0.2518 | 0.0022 | 0.0159 | 0 | 0.0007 | 0.6489 | 0.4419 | 0.0591 |
| 9604 | RNF14 | -1.12 | -0.35 | 1.89 | 1.33 | 0.17 | -0.55 | 1.06 | 1.77 | 3.42 | 3.93 | 1.08 | 0.21 | 0 | 0.6669 | 0.0121 | 0.3425 | 0.9438 | 0.7307 | 0.6576 | 0.0141 | 0.016 | 0.0038 | 0.6765 | 0.9049 |
| 54476 | RNF216 | -1.18 | -1.96 | 0.18 | -0.78 | 0.42 | -1.85 | -2.38 | -4.41 | -3.7 | -0.52 | 0.13 | -1.76 | 0.0008 | 0.001 | 0.7854 | 0.3978 | 0.5587 | 0.0585 | 0.1685 | 0 | 0 | 0.4095 | 0.9033 | 0.1434 |
| 4736 | RPL10A | -0.78 | -2.3 | -0.34 | 4.07 | 0.56 | 0.08 | -0.24 | -0.89 | -2.33 | -0.64 | 1.13 | -1.5 | 0.2275 | 0.2456 | 0.8486 | 0.3208 | 0.8133 | 0.9051 | 0.3928 | 0.3399 | 0.0009 | 0.5771 | 0 | 0.7396 |
| 6161 | RPL32 | -2.42 | -3.87 | -2.7 | -0.52 | -0.82 | -2.29 | -2.93 | -2.39 | -2 | 0.64 | -1.56 | -0.88 | 0 | 0 | 0.0076 | 0.7068 | 0.4794 | 0 | 0.0104 | 0 | 0.0006 | 0.2744 | 0 | 0.6415 |
| 25873 | RPL36 | -1.66 | -1.77 | -3.51 | -4.13 | -4.11 | -1.63 | -2.06 | -1.65 | -2.85 | 2.04 | 2.35 | -0.7 | 0.0007 | 0.0638 | 0.0003 | 0.0023 | 0 | 0.0651 | 0.0106 | 0.0156 | 0.0002 | 0.095 | 0.0426 | 0.6072 |
| 116832 | RPL39L | -3.09 | -0.98 | 0.52 | -2.56 | -0.65 | -0.05 | 1.18 | 1.15 | -2.54 | 3.1 | 1.9 | -2.01 | 0.0217 | 0.2768 | 0.8042 | 0.4431 | 0.8195 | 0.9795 | 0.6887 | 0.0041 | 0 | 0.2166 | 0.4256 | 0.3523 |
| 285855 | RPL7L1 | -2.87 | -0.08 | -0.26 | -1.29 | -1.75 | -2.96 | -2.36 | -2.73 | -1.08 | -1.57 | -0.89 | -0.61 | 0.0015 | 0.9354 | 0.8471 | 0.5055 | 0.1094 | 0.0005 | 0.0423 | 0 | 0.4581 | 0 | 0.5471 | 0.8241 |
| 400156 | RPS26L | 2.06 | 2.81 | 1.46 | 2.17 | 0.08 | 0.42 | -0.86 | -1.17 | -2.21 | -0.09 | 0.53 | -0.39 | 0.1387 | 0.0703 | 0.0092 | 0.5427 | 0.9397 | 0.721 | 0.7178 | 0.0757 | 0.0676 | 0.9454 | 0.8814 | 0.6809 |
| 51065 | RPS27L | 2.96 | 3.26 | 5.78 | 5.69 | 3.8 | 3.24 | 2.96 | 4.51 | 3.85 | 4.82 | 4.08 | 3.28 | 0 | 0 | 0 | 0.0001 | 0.0653 | 0.0002 | 0.1412 | 0 | 0.0011 | 0 | 0.0622 | 0.0006 |
| 6241 | RRM2 | -0.83 | -0.31 | 0.38 | -0.28 | -0.72 | -2 | -3.18 | -3.39 | -0.71 | -1.66 | -1.68 | -1.71 | 0.1039 | 0.2587 | 0.5956 | 0.7576 | 0.345 | 0.0005 | 0.0327 | 0 | 0.5592 | 0 | 0.3665 | 0.3452 |
| 345895 | RSHL3 | 0.51 | 0.67 | 0.34 | 0.19 | -0.24 | 0.52 | -0.01 | 0.43 | 0.36 | -0.72 | -0.22 | 0.44 | 0.058 | 0.0047 | 0.2889 | 0.7532 | 0.0276 | 0.233 | 0.9536 | 0.046 | 0.0217 | 0.0247 | 0.3784 | 0.258 |
| 22955 | SCMH1 | -1.28 | -0.98 | -3.39 | -4.46 | -3.28 | -2.86 | -2.2 | -1.16 | -0.87 | 1.31 | -1.04 | -1.82 | 0 | 0.0002 | 0 | 0.0237 | 0.0003 | 0.0016 | 0.0003 | 0.114 | 0.2256 | 0.339 | 0.4299 | 0.1187 |
| 6509 | SLC1A4 | -1.17 | -0.01 | -1.65 | -0.54 | -0.12 | -0.47 | -1.47 | -3 | -2.56 | -1.11 | 0.92 | -2.55 | 0.0222 | 0.96 | 0.0244 | 0.4731 | 0.8694 | 0.4999 | 0.0302 | 0 | 0.0004 | 0.1142 | 0.1599 | 0 |
| 6541 | SLC7A1 | -1.62 | -1.75 | 0.12 | -1.24 | -0.92 | -3.52 | -2.76 | -2.15 | -0.26 | -1.85 | -2.97 | -1.84 | 0.0505 | 0.1806 | 0.9162 | 0.1888 | 0.3133 | 0.0027 | 0.0223 | 0.0252 | 0.74 | 0.0233 | 0 | 0.6687 |
| 10650 | SLMO1 | -1.66 | -3.54 | -4.33 | -1.51 | -6.26 | -3.86 | -2.66 | -2.34 | -2.87 | 1.25 | -0.14 | -1.72 | 0.0017 | 0.0001 | 0.0179 | 0.5477 | 0.0015 | 0.0043 | 0.049 | 0.0002 | 0.0041 | 0.4388 | 0.9009 | 0.2727 |
| 57228 | SMAGP | -3.8 | -3.71 | -1.83 | -2.51 | -4.43 | -1.96 | -2.23 | -1.93 | -4 | 2.61 | -0.39 | -1.58 | 0 | 0.0022 | 0.277 | 0.1416 | 0 | 0.0285 | 0.0967 | 0.0145 | 0.0015 | 0.1751 | 0.725 | 0.0229 |
| 6601 | SMARCC2 | -0.97 | -1.91 | -2 | -1.64 | -1.56 | -1.06 | -2.29 | -4.14 | -6.09 | -2.37 | -3.04 | -1.7 | 0.073 | 0.0723 | 0.0001 | 0.3284 | 0.3931 | 0.4975 | 0.0769 | 0.004 | 0.0038 | 0.104 | 0.3326 | 0.1164 |
| 6625 | SNRP70 | 0.36 | -0.01 | -0.79 | 0.37 | -1 | -0.65 | -1.53 | -1.77 | -3.04 | -3.32 | -0.04 | 0.31 | 0.7833 | 0.9844 | 0.172 | 0.8045 | 0.4318 | 0.4527 | 0.0186 | 0.057 | 0.0025 | 0.0328 | 0.9858 | 0.6981 |
| 8405 | SPOP | -3.02 | -2.38 | -1.9 | -3.99 | -4.33 | -4.91 | -3.63 | -2.72 | -1.34 | -0.68 | -1.8 | -1.46 | 0 | 0.0253 | 0.15 | 0 | 0.0079 | 0 | 0.0401 | 0.0028 | 0.2386 | 0.4098 | 0.4113 | 0.4343 |
| 57522 | SRGAP1 | -1.53 | -1.77 | -1.27 | -2.42 | -0.93 | -1.92 | -2.56 | -3.49 | -3.07 | -2.36 | -1.07 | -2.99 | 0.1642 | 0.0802 | 0.1687 | 0.0476 | 0.4356 | 0.0253 | 0.0283 | 0 | 0.0006 | 0 | 0.5579 | 0.0965 |
| 23524 | SRRM2 | -1.22 | 0.12 | 0.86 | -0.61 | -1.5 | -1.3 | -3.07 | -2.39 | -4.49 | -4 | 0.5 | -0.91 | 0.4552 | 0.9329 | 0.4358 | 0.5237 | 0.1902 | 0.5014 | 0.0624 | 0 | 0 | 0.0067 | 0.7048 | 0.824 |
| 6776 | STAT5A | -1.44 | -1.52 | 1.19 | -0.72 | -0.94 | -1.85 | -1.79 | -2.15 | -3.45 | -1.28 | -1.6 | -1.39 | 0.0589 | 0.0674 | 0.0014 | 0.4439 | 0.3244 | 0.1207 | 0.1423 | 0 | 0.0137 | 0.1579 | 0.1682 | 0.0651 |
| 6789 | STK4 | -1.43 | -0.12 | 1.98 | -1.76 | -1.03 | -3.59 | -3.98 | -2.39 | -0.67 | 0.16 | 0.18 | 0.28 | 0.1676 | 0.9066 | 0.0544 | 0.0517 | 0.4399 | 0.0327 | 0.0126 | 0.0083 | 0.4448 | 0.8525 | 0.8678 | 0.8913 |
| 83931 | STK40 | -1.17 | -1.4 | -0.27 | 0.08 | -3.52 | -0.98 | -2.69 | -2.5 | -3.84 | 2.72 | 1.52 | -0.68 | 0.0974 | 0.1741 | 0.3737 | 0.9805 | 0.2725 | 0.6247 | 0.1725 | 0.0206 | 0.039 | 0.3414 | 0.3484 | 0.3386 |
| 6612 | SUMO3 | -1.9 | -1.78 | -2.79 | 1.71 | -1.96 | -1.55 | -3.76 | -2.62 | -2.93 | 1.85 | -1.68 | -0.91 | 0.1391 | 0.0262 | 0.3528 | 0.5571 | 0.0292 | 0.0071 | 0.0402 | 0.0058 | 0.0218 | 0.5418 | 0.5423 | 0.0346 |
| 10460 | TACC3 | -1.3 | -1.06 | 0.21 | -1.55 | -1.37 | -1.22 | -2.74 | -4.42 | -3.52 | -0.78 | -1.5 | -1.02 | 0.0595 | 0.1702 | 0.8808 | 0.0377 | 0.2352 | 0.2128 | 0.0233 | 0 | 0.0001 | 0.0202 | 0.218 | 0.0722 |
| 6895 | TARBP2 | -1.56 | -1.73 | 3.95 | 0.99 | 1.39 | 3.21 | 1.35 | 2.89 | -0.43 | 2.1 | 2.41 | 1.31 | 0.0059 | 0.0247 | 0.2711 | 0.7958 | 0.2547 | 0.0003 | 0.2494 | 0.011 | 0.5981 | 0.0113 | 0 | 0.1174 |
| 6942 | TCF20 | -1.51 | 1.78 | 0.27 | -1.75 | 0.86 | -1.74 | -2.46 | -3.51 | -2.76 | -1.88 | 0.28 | -2.47 | 0.1888 | 0.0245 | 0.5176 | 0.0035 | 0.2355 | 0.1454 | 0.004 | 0.0002 | 0.031 | 0.1025 | 0.4706 | 0.0886 |
| 9392 | TGFBRAP1 | -3.21 | -1.34 | -0.73 | -3.05 | -0.57 | -1.56 | -2.27 | -3.19 | -1.37 | -1.67 | -0.67 | -0.64 | 0.0072 | 0.0614 | 0.2009 | 0.0003 | 0.5492 | 0.0386 | 0.0308 | 0 | 0.2291 | 0.0007 | 0.6744 | 0.5606 |
| 51497 | TH1L | -1.95 | -2.34 | -1.8 | -2.65 | -2.7 | -3.55 | -3.8 | -3.02 | -0.36 | -2.23 | -3.08 | -1.03 | 0.0007 | 0.0478 | 0.0517 | 0.0002 | 0.0186 | 0.0001 | 0.0015 | 0 | 0.5147 | 0.0703 | 0.0024 | 0.083 |
| 83660 | TLN2 | -1.17 | -1.36 | -1.18 | -0.27 | -0.92 | -0.55 | -1 | -1.09 | -2.54 | -1.69 | -1.33 | -2.95 | 0.0055 | 0.0015 | 0.0011 | 0.181 | 0.0135 | 0.0335 | 0.1456 | 0.0188 | 0.0252 | 0.0445 | 0.0986 | 0 |
| 219854 | LOC219854 | -2.68 | -4.1 | -1.13 | -2.12 | -1.96 | -2.67 | -1.1 | -1.3 | -2.92 | 1.04 | -0.18 | -0.67 | 0 | 0 | 0.5432 | 0.0732 | 0.1883 | 0.0003 | 0.1127 | 0.0319 | 0 | 0.3604 | 0.7068 | 0.2946 |
| 55116 | TMEM39B | -2.33 | -1.9 | -1.88 | -1.4 | -2.55 | -1.78 | -2.82 | -3.26 | -3.85 | -0.14 | -1.31 | -2.21 | 0 | 0.0071 | 0 | 0.2151 | 0.0039 | 0.1288 | 0.0421 | 0.003 | 0.0243 | 0.8847 | 0.3848 | 0.1797 |
| 83460 | TMEM93 | -2.51 | -2.05 | 0.79 | -1.41 | -1.7 | 0.99 | 2.73 | 3.13 | -2.13 | 3.92 | 6.58 | -0.39 | 0.0237 | 0.2103 | 0.7292 | 0.5976 | 0.4508 | 0.6605 | 0.3434 | 0.0034 | 0.014 | 0.0981 | 0 | 0.8996 |
| 94241 | TP53INP1 | 0.8 | 1.67 | 2.69 | 3.2 | 3.69 | 3.86 | 4.4 | 3.11 | 2.1 | 2.84 | 3.21 | 1.38 | 0.2528 | 0.0006 | 0.0043 | 0.0003 | 0.0002 | 0.0087 | 0.028 | 0 | 0 | 0 | 0.0089 | 0.1563 |
| 7706 | TRIM25 | -2.44 | -2.05 | -1.96 | -3.07 | -2.09 | -1.43 | -0.81 | -2.68 | -3.93 | -3.89 | -1.36 | -2.15 | 0 | 0.0001 | 0.0061 | 0.0001 | 0 | 0 | 0.0006 | 0 | 0.0008 | 0.0535 | 0.4765 | 0.0001 |
| 81786 | TRIM7 | -2.71 | -2.84 | -0.58 | -0.52 | -3.09 | -2.29 | -3.25 | -3.38 | -3.81 | -0.53 | -1.09 | -1.61 | 0 | 0 | 0.3426 | 0.2616 | 0.0194 | 0.0038 | 0.0128 | 0 | 0 | 0.4683 | 0.0237 | 0.2454 |
| 10024 | TROAP | -1.05 | -4.24 | -4.42 | -1.07 | -5.18 | -2.85 | -3.94 | -4.44 | -4.77 | -1.1 | -3.22 | -0.02 | 0.3127 | 0 | 0.0083 | 0.7039 | 0.0097 | 0.1642 | 0.0326 | 0.0021 | 0.0046 | 0.4471 | 0.0626 | 0.9799 |
| 10099 | TSPAN3 | -3.22 | -1.07 | -1.43 | -2.42 | -1.87 | -2.62 | -2.52 | -1.66 | -0.96 | 0.54 | -0.35 | -2.68 | 0 | 0.3284 | 0.4251 | 0.0992 | 0.0005 | 0 | 0.0371 | 0.0143 | 0.1885 | 0.4433 | 0.6569 | 0.042 |
| 6302 | TSPAN31 | -0.32 | -0.85 | 1.91 | 1.23 | 1.43 | 1.59 | 1.96 | 3.89 | 1.12 | 2.92 | 2.44 | 1.65 | 0.4922 | 0.2739 | 0.0125 | 0.4332 | 0.272 | 0 | 0.0004 | 0.0001 | 0.0344 | 0.0006 | 0 | 0 |
| 150465 | TTL | -3.08 | -1.95 | -0.11 | -2.22 | -1.83 | -2.85 | -4.19 | -3.26 | -1.38 | 0.95 | -0.79 | -1.74 | 0.0001 | 0.0224 | 0.9215 | 0.1762 | 0.3493 | 0.016 | 0.021 | 0 | 0.139 | 0.6141 | 0.7371 | 0.1322 |
| 51060 | TXNDC12 | -0.44 | -0.52 | -3.88 | -2.02 | -2.04 | -2.86 | -1.7 | -1.95 | 1.46 | 0.63 | -2.2 | -1.26 | 0.69 | 0.3608 | 0.0345 | 0.0916 | 0.0432 | 0.0007 | 0.0472 | 0 | 0.0608 | 0.6343 | 0.3998 | 0.456 |
| 7322 | UBE2D2 | -2.11 | -2.78 | -3.48 | -3.05 | -4.2 | -3.26 | -2.65 | -1.62 | -0.71 | 0.92 | -0.24 | -1.95 | 0 | 0 | 0.0056 | 0.0326 | 0.009 | 0.0003 | 0.0286 | 0 | 0.1109 | 0.4434 | 0.7899 | 0 |
| 29855 | UBN1 | -2.61 | 0.85 | 0.23 | -1.07 | -1.64 | -3.16 | -3.87 | -3.65 | -2.08 | -3 | -1.8 | -1.86 | 0 | 0.0862 | 0.8097 | 0.0129 | 0.0036 | 0.0063 | 0.0006 | 0 | 0.0538 | 0.0001 | 0.0878 | 0.4733 |
| 7343 | UBTF | -1.12 | -0.42 | -0.66 | -0.52 | -0.64 | -0.7 | -1.13 | -2.01 | -3.1 | -1.24 | 1.1 | -1.21 | 0.0061 | 0.357 | 0.3888 | 0.3272 | 0.5325 | 0.2686 | 0.1168 | 0.0024 | 0.0012 | 0.0003 | 0.3408 | 0.0073 |
| 5976 | UPF1 | 0.06 | 0.38 | 0.84 | -0.08 | -0.73 | 0.22 | -0.54 | -1.06 | -3.16 | -2.97 | 0.62 | -1.46 | 0.9097 | 0.4475 | 0.3513 | 0.8565 | 0 | 0.569 | 0.1455 | 0.0096 | 0.0349 | 0.1012 | 0.682 | 0.2292 |
| 7398 | USP1 | -2.64 | -3.73 | -1.76 | -3.98 | -2.32 | -4.4 | -3.03 | -0.65 | 3.24 | 0.11 | -2.09 | -2.06 | 0 | 0.03 | 0.0192 | 0.0155 | 0.3728 | 0.1112 | 0.051 | 0.6702 | 0.0883 | 0.6186 | 0.4608 | 0.055 |
| 8237 | USP11 | -0.62 | -0.24 | -0.84 | -1.51 | -0.61 | 0.12 | -0.11 | -1.54 | -5.84 | -1.78 | -0.2 | -0.61 | 0.0273 | 0.7384 | 0.0218 | 0.0033 | 0.2383 | 0.9262 | 0.8779 | 0.2293 | 0 | 0.0039 | 0.8708 | 0.3273 |
| 57602 | USP36 | 0.36 | 1.43 | 4.52 | 2.42 | 4.64 | 1.78 | 0.48 | 0.22 | 0.55 | 3.36 | 2.19 | 0.87 | 0.6774 | 0.0056 | 0.0001 | 0.0055 | 0 | 0.0008 | 0.6336 | 0.6311 | 0.2858 | 0.0006 | 0 | 0.4473 |
| 7428 | VHL | -2.48 | -1.76 | -1.93 | -3.52 | -1.05 | -3.66 | -3.62 | -2.5 | -3.62 | 0.88 | 0.96 | -2.26 | 0.0149 | 0.0958 | 0.0232 | 0.0001 | 0.3681 | 0.0238 | 0.0313 | 0 | 0 | 0.4597 | 0.6818 | 0.0067 |
| 7430 | VIL2 | -1.36 | -0.68 | -0.54 | -3.07 | -1.42 | -1.35 | -2.33 | -2.81 | -0.89 | -3.4 | -2.04 | -1.78 | 0.0005 | 0.2546 | 0.8317 | 0.0311 | 0.0005 | 0 | 0.0425 | 0 | 0.319 | 0.1549 | 0.4171 | 0.6604 |
| 51652 | VPS24 | -3.06 | -4.18 | -4.17 | -6.86 | -4.64 | -4.51 | -4.13 | -3.82 | -3.24 | -0.27 | -0.64 | -2.44 | 0 | 0 | 0 | 0 | 0.0079 | 0 | 0 | 0 | 0 | 0.8256 | 0.5149 | 0.042 |
| 51652 | VPS24 | -2.76 | -2.28 | -4.47 | -6.76 | -4.99 | -4.41 | -3.68 | -4.42 | -2.84 | 0.11 | -1.55 | -2.89 | 0 | 0 | 0.0001 | 0 | 0.0005 | 0 | 0.0013 | 0 | 0 | 0.9452 | 0.0106 | 0.0396 |
| 11180 | WDR6 | -0.69 | -0.5 | 0.16 | -1.51 | -2.34 | -2.05 | -3.06 | -3.15 | -1.84 | -2.51 | -4.55 | -1.46 | 0.0064 | 0.1272 | 0.9114 | 0.1947 | 0.0803 | 0.0757 | 0.0007 | 0.0008 | 0.3884 | 0.2638 | 0.0107 | 0.5151 |
| 284273 | ZADH2 | -2.6 | -1.62 | -0.03 | -2.41 | -0.46 | -1.52 | -1.97 | -3.68 | -2.62 | -2.33 | -1.32 | -1.88 | 0.0086 | 0.0037 | 0.9221 | 0 | 0.4223 | 0.0487 | 0.007 | 0.0001 | 0.0912 | 0 | 0.281 | 0.137 |
| 9189 | ZBED1 | 4.14 | 4.9 | 7.56 | 2.88 | 0.84 | -0.34 | 2.28 | 4.27 | 6.01 | 2.18 | 2.37 | 4 | 0 | 0 | 0.0005 | 0.3327 | 0.3833 | 0.5242 | 0.0018 | 0 | 0 | 0.0093 | 0.0538 | 0.0384 |
| 80829 | ZFP91 | -2.88 | -4.88 | -0.13 | -2.77 | 0.03 | -3.43 | -3.01 | -1.61 | 0.82 | 0.1 | -1.64 | -1.69 | 0.0211 | 0.0002 | 0.9279 | 0 | 0.9868 | 0.0035 | 0.0323 | 0.0016 | 0.3514 | 0.8894 | 0.3578 | 0 |
| 10793 | ZNF273 | 1.24 | 0.32 | -0.26 | 0.28 | 1.22 | 2.6 | 4.08 | 1.68 | 1.67 | -1.86 | -2.05 | 0.35 | 0 | 0.4532 | 0.5882 | 0.7995 | 0.4639 | 0.1085 | 0.0079 | 0.1079 | 0 | 0.1079 | 0.0261 | 0.0012 |
| 149076 | ZNF362 | -2.13 | -0.29 | -0.41 | -3.48 | -2.21 | -1.06 | -1.93 | -2.72 | -3.52 | 1.01 | 1.34 | -1.33 | 0 | 0.4586 | 0.8172 | 0.0003 | 0.0527 | 0.2759 | 0.0006 | 0 | 0 | 0.3017 | 0.0016 | 0.0094 |
| 27300 | ZNF544 | -1.86 | -1.68 | -0.04 | -2.97 | -1.67 | -3.57 | -3.37 | -1.64 | -0.74 | 0.65 | -0.76 | -1.3 | 0.002 | 0.0421 | 0.971 | 0.0319 | 0.2062 | 0 | 0.002 | 0.0069 | 0.6217 | 0.6128 | 0.6527 | 0.4897 |
