# Supplementary material for: Down-Regulation of eIF4GII by miR-520c-3p Represses Diffuse Large B Cell Lymphoma Development
Source: PLoS Genet. 2014 Jan 30;10(1):e1004105. doi: 10.1371/journal.pgen.1004105 (PMC3907297; doi:10.1371/journal.pgen.1004105)
Supplement: Table S3 — eIF4GII (IHC staining) and miR-520c-3p (measured by RT-qPCR) expression in TMA of primary DLBCL samples. 0 - negative staining, 1 - positive staining. (DOCX) [file pgen.1004105.s011.docx]

**Table S3**

| **Sample ID** | **Tissue type** | **EIF4GII expression** | **miR-520c-3p levels (fold)** |
| --- | --- | --- | --- |
| 1 | Normal GCB | 0 | 0.93 |
| 2 | Normal GCB | 0 | 0.22 |
| 3 | Normal GCB | 0 | 1.00 |
| 4 | Normal GCB | 0 | 2.57 |
| 5 | Normal GCB | 0 | 4.37 |
| 6 | Normal GCB | 0 | 1.34 |
| 7 | DLBCL | 0 | 0.00 |
| 8 | DLBCL | 0 | 0.43 |
| 10 | DLBCL | 0 | 0.02 |
| 11 | DLBCL | 0 | 0.70 |
| 12 | DLBCL | 0 | 0.04 |
| 13 | DLBCL | 0 | 5.53 |
| 14 | DLBCL | 0 | 0.06 |
| 15 | DLBCL | 0 | 1.09 |
| 16 | DLBCL | 0 | 0.24 |
| 17 | DLBCL | 0 | 1.05 |
| 18 | DLBCL | 0 | 9.39 |
| 19 | DLBCL | 0 | 0.03 |
| 20 | DLBCL | 0 | 4.77 |
| 21 | DLBCL | 0 | 0.06 |
| 22 | DLBCL | 1 | 0.25 |
| 23 | DLBCL | 1 | 0.03 |
| 24 | DLBCL | 1 | 0.01 |
| 25 | DLBCL | 1 | 0.02 |
| 26 | DLBCL | 1 | 0.38 |
| 27 | DLBCL | 1 | 0.74 |
| 28 | DLBCL | 1 | 0.55 |
| 29 | DLBCL | 1 | 0.05 |
| 30 | DLBCL | 1 | 0.17 |
| 31 | DLBCL | 1 | 0.07 |
| 32 | DLBCL | 1 | 0.09 |
| 33 | DLBCL | 1 | 0.10 |
| 34 | DLBCL | 1 | 0.23 |
| 35 | DLBCL | 1 | 1.69 |
| 36 | DLBCL | 1 | 0.30 |
| 37 | DLBCL | 1 | 0.17 |
| 38 | DLBCL | 1 | 0.16 |
| 39 | DLBCL | 1 | 0.11 |
| 40 | DLBCL | 1 | 0.23 |
| 41 | DLBCL | 1 | 0.36 |
| 42 | DLBCL | 1 | 0.02 |
| 43 | DLBCL | 1 | 0.00 |
| 44 | DLBCL | 1 | 0.56 |
| 45 | DLBCL | 1 | 1.18 |
| 46 | DLBCL | 1 | 0.04 |
| 47 | DLBCL | 1 | 3.03 |
| 48 | DLBCL | 1 | 0.16 |
| 49 | DLBCL | 1 | 0.01 |
| 50 | DLBCL | 1 | 0.06 |
| 51 | DLBCL | 1 | 0.22 |
| 52 | DLBCL | 1 | 0.53 |
| 53 | DLBCL | 1 | 0.38 |
| 54 | DLBCL | 1 | 0.74 |
