# Supplementary material for: Down-Regulation of eIF4GII by miR-520c-3p Represses Diffuse Large B Cell Lymphoma Development
Source: PLoS Genet. 2014 Jan 30;10(1):e1004105. doi: 10.1371/journal.pgen.1004105 (PMC3907297; doi:10.1371/journal.pgen.1004105)
Supplement: Table S4 — EpiTYPER primer sequences designed for miR520c. (DOC) [file pgen.1004105.s012.doc]

**Table S4**

| **Primer** | **Sequence** |
| --- | --- |
| | MA520C-1F | | --- | | MA520C-1R | | | aggaagagagGGTTTTTTTAATTTAGATTTTTTGGG | | --- | | cagtaatacgactcactatagggagaaggctAAAACACAACAACTCACACCACTAA | |
| | MA520C-2F | | --- | | MA520C-2R | | | aggaagagagTTAGTGGTGTGAGTTGTTGTGTTTT | | --- | | cagtaatacgactcactatagggagaaggctACAATCCTCCTAAATTTTATTCCCA | |
| | MA520C-3F | | --- | | MA520C-3R | | | aggaagagagAGGGATTTATGTTTTGGATTTTAGA | | --- | | cagtaatacgactcactatagggagaaggctTTTTAAAAACCCTATCTCAAAAAAA | |
| | MA520C-4F | | --- | | MA520C4R | | | aggaagagagTTTTTATATTATTTTGGGGTTGTTT | | --- | | cagtaatacgactcactatagggagaaggctACTCCTCAATAACATTTATACAAAAATAC | |
| | MA520C-5F | | --- | | MA520C-5R | | | aggaagagagGATTTTTAAAGTGTTGGGATTAGAGG | | --- | | cagtaatacgactcactatagggagaaggctTCCTCCTAAATTTTATCTCCAAAAA | |
