# Supplementary material for: Down-Regulation of eIF4GII by miR-520c-3p Represses Diffuse Large B Cell Lymphoma Development
Source: PLoS Genet. 2014 Jan 30;10(1):e1004105. doi: 10.1371/journal.pgen.1004105 (PMC3907297; doi:10.1371/journal.pgen.1004105)
Supplement: Text S1 — Single Locus Quantitative DNA Methylation Assays. (DOC) [file pgen.1004105.s013.doc]

Text S1.

Mass Array Sequenom Epityping (Sequenom, CA) was performed on bisulfite-converted DNA. High molecular weight DNA was isolated from normal reactive B cells (GCB=Germinal Center B cells purified from reactive human tonsillar tissue), DLBCL cell lines and primary DLBCL cases. EpiTYPER primers were designed to cover the coding sequence of miR520c and up to 1kB upstream and downstream from it by using Sequenom EpiDesigner beta software (http://www.epidesigner.com/) (Table S3).
